# Genome-wide expression analysis reveals involvement of asparagine synthetase family in cotton development and nitrogen metabolism

**DOI:** 10.1186/s12870-022-03454-7

**Published:** 2022-03-16

**Authors:** Asif Iqbal, Gui Huiping, Wang Xiangru, Zhang Hengheng, Zhang Xiling, Song Meizhen

**Affiliations:** 1 State Key Laboratory of Cotton Biology, Institute of Cotton Research of Chinese Academy of Agricultural Sciences, Zhengzhou Research Base, School of Agricultural Sciences, Zhengzhou University, State Key Laboratory of Cotton Biology, Anyang, Henan 455000 People’s Republic of China; 2 Western Agricultural Research Center of Chinese Academy of Agricultural Sciences, Changji, 831100 Xinjiang China

**Keywords:** Cotton, *Gossypium hirsutum*, Asparagine synthetase, Bioinformatics analysis, Nitrogen metabolism

## Abstract

Asparagine synthetase (ASN) is one of the key enzymes of nitrogen (N) metabolism in plants. The product of ASN is asparagine, which is one of the key compounds involved in N transport and storage in plants. Complete genome-wide analysis and classifications of the ASN gene family have recently been reported in different plants. However, little is known about the systematic analysis and expression profiling of ASN proteins in cotton development and N metabolism. Here, various bioinformatics analysis was performed to identify ASN gene family in cotton. In the cotton genome, forty-three proteins were found that determined ASN genes, comprising of 20 genes in *Gossypium hirsutum* (Gh), 13 genes in *Gossypium arboreum*, and 10 genes in *Gossypium raimondii*. The ASN encoded genes unequally distributed on various chromosomes with conserved glutamine amidotransferases and ASN domains. Expression analysis indicated that the majority of GhASNs were upregulated in vegetative and reproductive organs, fiber development, and N metabolism. Overall, the results provide proof of the possible role of the ASN genes in improving cotton growth, fiber development, and especially N metabolism in cotton. The identified hub genes will help to functionally elucidate the ASN genes in cotton development and N metabolism.

**Supplementary Information:**

The online version contains supplementary material available at 10.1186/s12870-022-03454-7.

## Background

Nitrogen (N) metabolism is one of the most important plant metabolic processes [1–5]. After uptake, N passes through various N metabolizing enzymatic activities to produce glutamine, glutamate, aspartate, and asparagine that are further used to produce various amino acids and N-containing compounds [6]. Among these enzymes, asparagine synthetase (ASN) is one of the most important enzymes responsible for the synthesis of asparagine from aspartate. Asparagine synthesis occurs by the amination of aspartate which is catalyzed by ASN in an ATP-dependent amidotransferase reaction [7]. The N and carbon ratio (2:4) of ASN is high and stable [8] that’s why acting as an efficient N transport and storage carrier, therefore, plays a key role in N metabolism of higher plants [9, 10]. ASNs can be classified into two categories according to their structural characteristics such as ammonium dependent ASN-A and glutamine dependent ASN-B [11]. There are three synthetic pathways of asparagine in plants among which the major one is regulated by ASN-B, where asparagine is produced from the reaction of ammonium or glutamine with aspartate. It was found that ASN-B in plants is mediated by light and metabolites related to plant development like vegetative organs and seed development [12]. In tuber crops, it was found that asparagine is responsible for the ASN-B synthesis and not in the transport from leaf to the tuber, confirming the role of ASN in N transport and metabolism [13]. Thus, ASN-B has an essential role in N metabolism, N transportation within the tissues, and redistribution of glutamate and glutamine in various plant organs [12].

In ASN, the amino acids sequences are comprised of two conserved domains such as the C-terminal synthetase domain and the glutamine amidotransferase domain (GATase) [14]. The GATase binds to glutamine encompasses from the N-terminal to the fourth amino acid position has the structural characteristics of Met-Cys-Gly-Ile [15, 16]. The three conserved sites of the ASN domain are Cyc, His, and Asp, which are located on the N-terminal of the polypeptide [16, 17]. These domains are involved in the transamination of glutamine [17]. Additionally, the adenosine monophosphate and aspartic acid are localized on the C-terminal [18]. The ASN encoded polypeptide is comprised of 579–591 amino acids having an approximate molecular weight of 65 kDa [19].

The ASN gene family of Arabidopsis is very simple and short with the functionally identified members such as AtASN1, AtASN2, and AtASN3 [12]. Additionally, an uncharacterized member (At2g03667) having an ASN domain is also discovered but its biological function is still to be elucidated. Studies have found that both ASN1 and ASN2 show common expression in the genes against light and N metabolites [20]. Similarly, in common bean and sunflower, the expression of these genes was inhibited [21, 22], probably due to interaction with the photosynthetic process. The expression of ASN1 is in line with the changes in the level of total free asparagine, however, the expression of ASN2 was higher in Arabidopsis vegetative tissues [23]. A previous study in Arabidopsis showed that the ASN1 gene is encoded by a glutamine-dependent member [15]. In Arabidopsis, ASN1 is involved in the N filling during seed development [6] and ASN2 contributes to the primary N metabolism in the vegetative organs [23]. Additionally, ASN2 is involved in the detoxification of ammonium through stress-induced expression [24]. Earlier, the expression of ASN genes was related to N form and content such as Arabidopsis AtASN2 was induced by ammonium, which also increase its expression level [24]. In other studies, *Phaseolus vulgaris* PvASN1 and PvASN2 [25] and soybean SASN1, SASN2, and SASN3 genes were induced by nitrate [26]. In cotton, asparagine is the main product of ASN that acts as a key compound for N transport and metabolism [4]. However, there is a lack of study on the characterization of the ASN gene family, its expression pattern, regulation mode and function in cotton.

Asparagine and glutamine are the most important form of N transport from root to shoot. In rice phloem sap, the major form of N transfer is asparagine and glutamine, while in soybean it is only asparagine [27]. In earlier studies, a high level of asparagine and amino acids were used for screening high protein genotypes [28–31]. ASN1 and ASN2 play a diverse role in rice, where OsASN1 is allocated to produce asparagine during ammonium assimilation in rice roots, while OsASN2 is involved in asparagine transport to other parts of the plant [32]. It was also found that ASN has an important role in germination because asparagine accumulated as predominant amide in the process of germination [33]. In *Medicago truncatula*, the leaves lost about 90% of chlorophyll at senescence, and the gene involved in ASN and glutamine synthetase were upregulated and the proteins and nucleic acid were dissociated into glutamine and asparagine. Consequently, these compounds were translocated for the formation of new tissues as well as grain [34, 35].

Recently, the deciphering of genome sequencing of many plant species had facilitated the comprehensive classification and functional analysis of various gene families [36]. So far, the detailed systematic analysis and expression profiling of ASN proteins in cotton has not been elucidated. Therefore, the current study aimed to evaluate the structural characterization of the ASN gene family and to provide the foundation for further functional analysis of key ASN genes and their roles in growth, development, and N metabolism in *Gossypium* (G) species. Moreover, the study will enrich the researchers to use the key ASN genes for advanced genome editing techniques to improve N use efficiency and develop N-efficient cotton genotype.

## Materials and methods

### Identification of ASN protein family members

For identification of the ASN family in cotton, the genome files of three cotton species such as *G. hirsutum*, *G. arboreum*, and *G. raimondii* were downloaded from the Cotton Functional Genomics Database (https://cottonfgd.org/) [37]. The genomic sequences of *Arabidopsis thaliana*, *Oryza sativa*, and *Theobroma cacao* were retrieved from phytozome (https://phytozome-next.jgi.doe.gov/). The domain analysis programs PFAM and SMART were used to examine the protein sequences of the collected ASN genes [38]. Further, the domain of all ASN proteins was searched in PFAM through the hidden Markov model [39]. The protein sequences with Pfam numbers of PF13537.1 and PF00733.16 were obtained, which comprised of ASN and GATase domain. Finally, the protein sequences of ASN were matched with the known sequences through ClustalX to confirm the protein sequences [40]. The amino acid length of the protein, molecular weight, charge, isoelectric points, and grand average of hydropathy was identified from cottonFGD.

### Phylogenetic analysis and sequence alignment

The full-length amino acid sequences of *G. hirsutum*, *G. arboreum*, *G. raimondii*, *Oryza sativa*, *Theobroma cacao*, and *Arabidopsis thaliana* encoded by ASN genes were aligned with the ClustalX program and were adjusted manually in MEGA7.0 [41]. To confirm the results of ClustalX, the multiple sequence alignment was also performed by using the MUSCLE program (ver. 3.52) [42]. Subsequently, the phylogenetic tree was built through the neighbor-joining (NJ) method with 1000 bootstrap replicates and default parameters in the MEGA7.0 program [43].

### Analysis of conserved motifs and gene structure

The domain analysis programs PFAM and SMART were used to examine the protein structures. The ASNs protein sequences of cotton were used to identify the conserved motifs through MEME analysis. The genome data downloaded from cottonFGD were used to construct gene structures by using the Gene Structure Display Server (http://gsds.cbi.pku.edu.cn/).

### Chromosomal locations and collinearity

Physical positions of chromosomal locations of the given ASN genes from cotton species were constructed in TBtools software [44]. The collinearity analysis was performed by using the GFF3 file, linked file, and gene IDs [44]. Subsequently, the collinearity between the homologous genes pairs was visualized by the circle gene viewer model of TBtools software [44].

### Selection pressure and promoter analysis

For estimation of the Ka/Ks ratio, the CDS of homologous gene pairs of three cotton species were used to analyze the selection pressure between ASN genes in each pair of the genome and subgenome using TBtools software [44]. The cis-regulatory elements in the promoter regions of GhASN genes were analyzed through the PlantCARE database using the upstream sequences (2000) of ASN proteins downloaded from cottonFGD [37].

### Expression profiling and gene co-expression network analysis

The expression profiling of ASN genes of *G. hirsutum* was calculated as fragments per kilobase of exon per million mapped (FPKM). The transcriptomic data (PRJNA248163 and PRJNA606910) from NCBI (National Center for Biotechnology Information) (https://www.ncbi.nlm.nih.gov/) were used to analyze differentially expressed ASN genes in vegetative and reproductive organs, fiber development [45], and N metabolism [4]. Based on the FPKM values, the heat map was generated through TBtool software. Moreover, to identify the hub genes in vegetative and reproductive organs, fiber development as well as in N metabolism, a co-expression network analysis was performed according to our previous study [4]. The obtained data were visualized in the Cytoscape software [46] in the form of a co-expression network. Subsequently, the hub genes were identified within the network according to the maximum number of positive correlations between the nodes [4].

## Results

### Genome-wide identification of cotton ASN proteins

An in silico search was accomplished to identify the ASN members in the cotton functional genomic database (cottonFGD) (www.cottonfgd.org) using the domain number PF13537.1 and PF00733.16. Subsequently, the ASN proteins of *G. hirsutum*, *G. arboreum*, and *G. raimondii* were retrieved from cottonFGD and those of *Oryza sativa*, *Theobroma cacao*, and *Arabidopsis thaliana* were downloaded from phytozome. Forty-three ASN encoded proteins were identified in the sequenced genome of all three cotton species, with 13, 10, and 20 ASN in *G. arboreum, G. raimondii,* and *G. hirsutum*, respectively. All the 20 genes encoded proteins ranging from 362 (*Ghir_A13G023660*) to 589 (*Ghir_D13G010010*) amino acids, with isoelectric points varying from 5.97 (*Ghir_A12G017120*) to 7.12 (*Ghir_A12G004130*), molecular weight varying from 40.35 (*Ghir_A13G023660*) kDa to 66.03 (*Ghir_A07G025290*) kDa, molecular charge ranged from − 6 (*Ghir_D09G009430*) to 7 (*Ghir_A05G008930*), and the grand average of hydropathy was − 0.39 (*Ghir_D12G017370*) to − 0.02 (*Ghir_D12G003150*) (Table S1). Among them, 11 ASN genes were positioned on At subgenome, and the rest were located on the Dt subgenome.

### Phylogenetic analysis

**Fig. 1:**
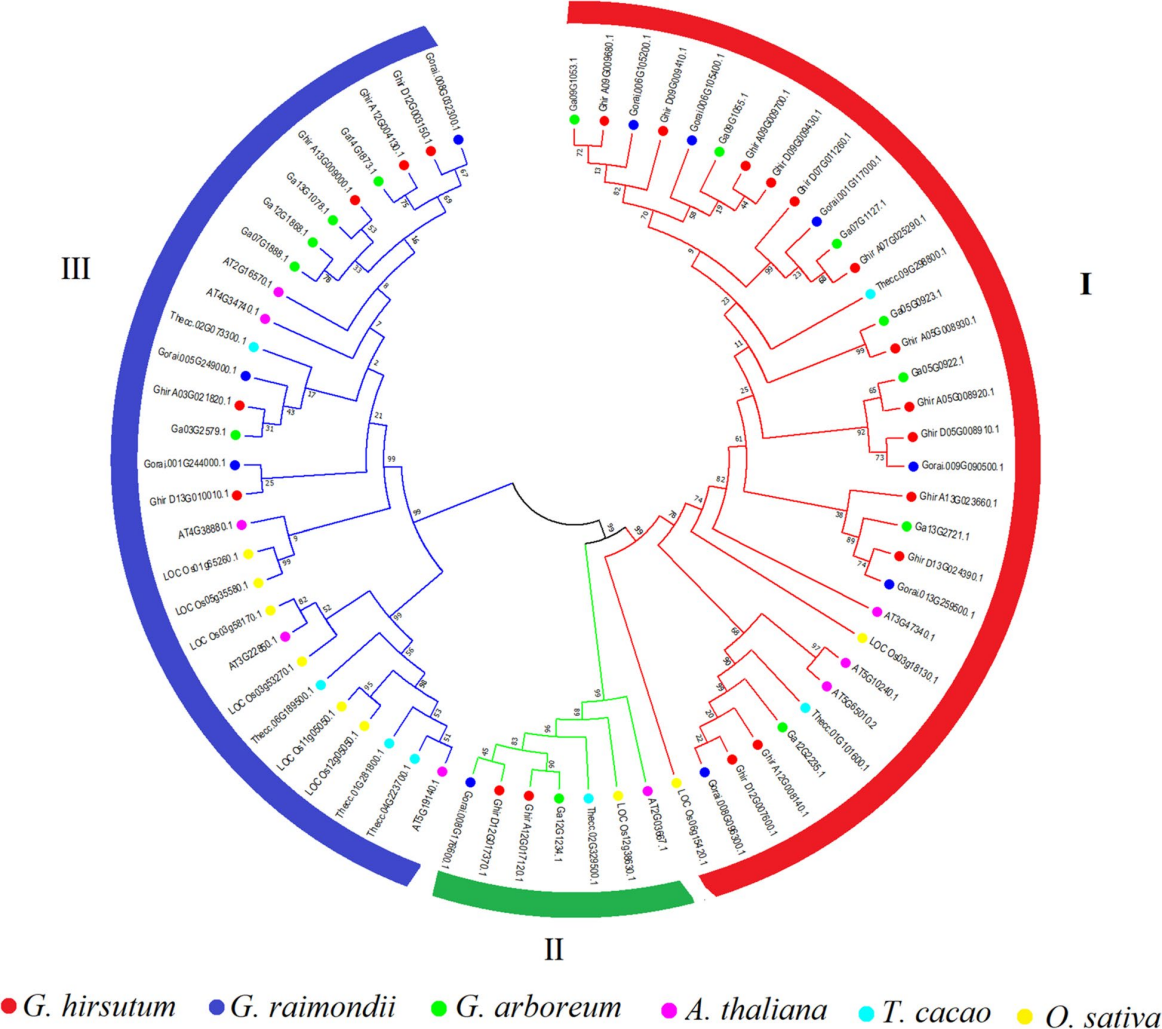
Phylogenetic analysis of ASN protein from *G. hirsutum*, *G. raimondii*, *G. arboreum*, *A. thaliana*, *T. cacao*, and *O. sativa*. I, II, and III indicate the three groups of the phylogenetic tree

The phylogenetic analysis of 61 ASN protein sequences (20 from *G. hirsutum*, 13 from *G. arboreum,* 10 from *G. raimondii,* 10 from *Arabidopsis thaliana*, 8 from *Theobroma cacao*, and 10 from *Oryza sativa*) was submitted to build an unrooted tree based on multiple sequence alignment using NJ method in MEGA7.0 (Fig. 1). The results suggested that ASN genes could be categorized into three subgroups such as group I, group II, and group III, and all the ASN genes were symmetrically distributed within the three cotton species in each group (Fig. 1), while in *Oryza sativa*, *Theobroma cacao*, and *Arabidopsis thaliana*, ASN genes were identified in cluster forms (Fig. 1). Therefore, cotton ASN members in their identified groups might be evolutionary close within respective species. Moreover, GhASN genes were identified in all three groups with the highest share in group I with 13 GhASNs, group II contains 2 GhASNs, and group III consists of 5 GhASNs. The results further demonstrated that group I having the highest number of ASN genes from all the selected plant species is an ancient group of ASN members. Unlike *Arabidopsis thaliana* and *Oryza sativa*, the ASN genes from cacao have a close relationship with cotton, as the genes of both species were clustered closely with each other in the subgroups of the phylogenetic tree (Fig. 1) supporting the hypothesis that cotton and cacao shared close and similar ancestors [47].

### Gene structure, domain architecture, and conserved motifs identification of ASN proteins

**Fig. 2:**
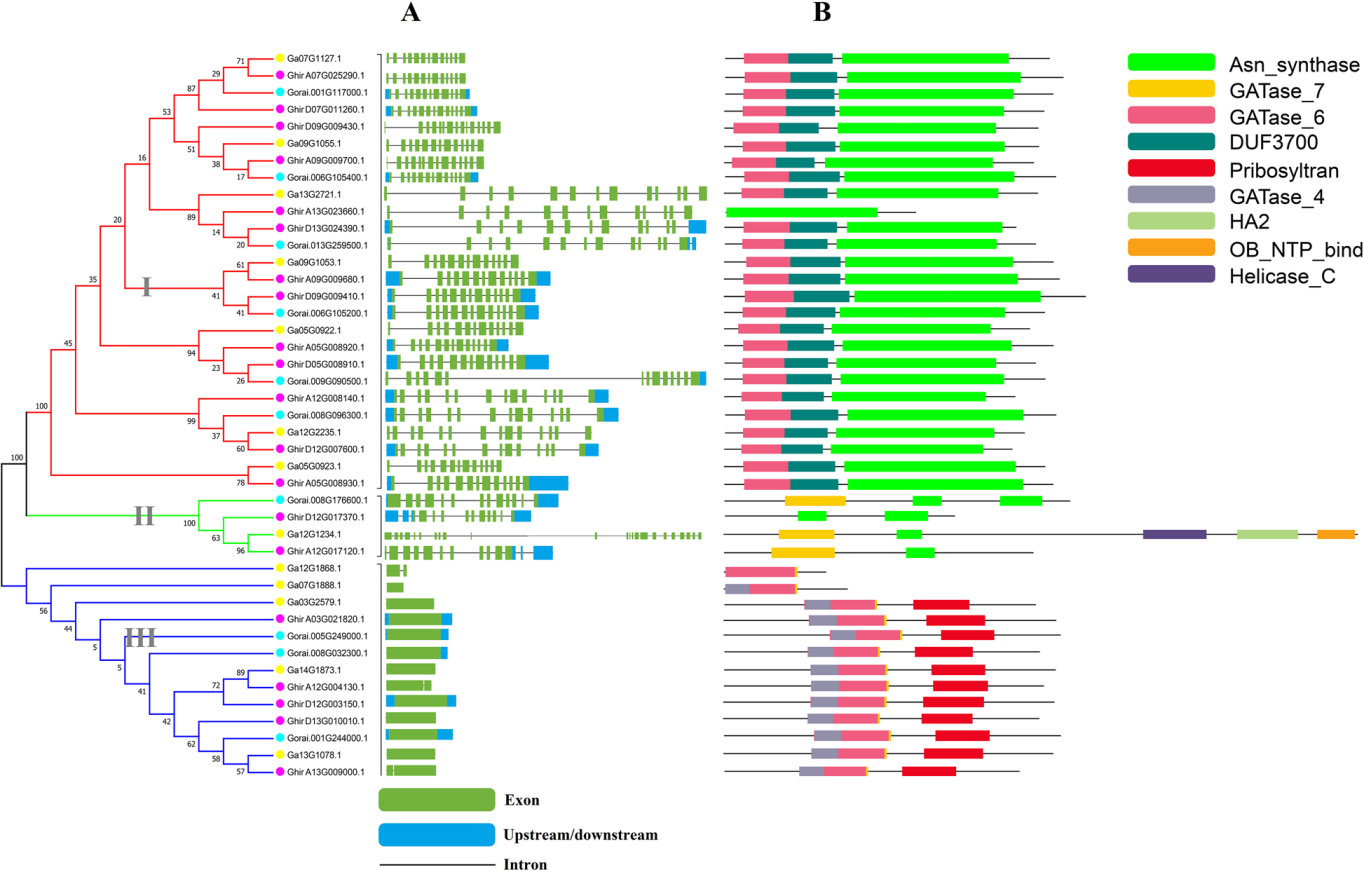
The phylogenetic tree of ASN protein three cotton species. **A** Gene structure display of ASN genes in three cotton species, where exon and UTR region are represented as a green and blue rectangular shape, respectively, while the black line shows intron. **B** Domain architecture of ASN genes

The exons and introns were analyzed to know the gene structure variation in the cotton ASN family. The results demonstrated that cotton ASN genes are highly conserved in structure. As expected, the ASN genes clustered within the subgroup of the phylogenetic analysis were found structurally alike (Fig. 2A). The ASN genes greatly varied in exon and intron number with one exon and no intron to the maximum of 23 exons and 22 introns (Fig. 2A). Two members in *G. raimondii* including *Gorai.009G09500.1* and *Gorai.008G096300.1* contain fourteen exons, while *G. hirsutum* and *G. arboreum* contains 15 (*Ghir_D0G009430.1*) and 23 (*Ga12G1234.1*) as highest exons, respectively (Fig. 2A). Among the subgroups, group I and group II have the highest exons of 15 and 23, while group III contains 1 or 2 exons (Fig. 2A). These results showed that ASN genes are structurally different according to their features.

Further, protein functional domain analysis was performed to understand the function of ASNs. As predicted, each subgroup has different domain compositions with ASN and GATase_6 domain occurred in the ASN members of group I. Group II contains GATase_7 and ASN domain, and group III is mainly composed of GATase_6 and a part of GATase_7. GATase_6 domain is a class-II glutamine amidotransferase domain found in asparagine synthetase and glutamine-fructose-6-phosphate transaminase. GATase_7 helps to regulate the removal of ammonium from glutamine and translocated it to produce new carbon and nitrogen molecules. However, the ASN domain helps to form asparagine from aspartate (Fig. 2B). In each group, the conserved motif was identified through the conserved motifs prediction and their respective logos (Fig. S1). The MEME analysis identified 20 different motifs in the three cotton species (Fig. S1). The structure of motifs was found similar according to the subgroup of the phylogenetic tree. The results revealed that motif 1, motif 2, and motif 3 were enriched in group I, motif 15 and motif 18 was enriched in group II, and motif 13, motif 16, and motif 17 were enriched in group III (Fig. S1). As expected, the protein motifs within the same group of ASNs were found similar (Fig. S1).

### Chromosomal localization, collinearity, and selection pressure analysis of the ASN genes

**Fig. 3:**
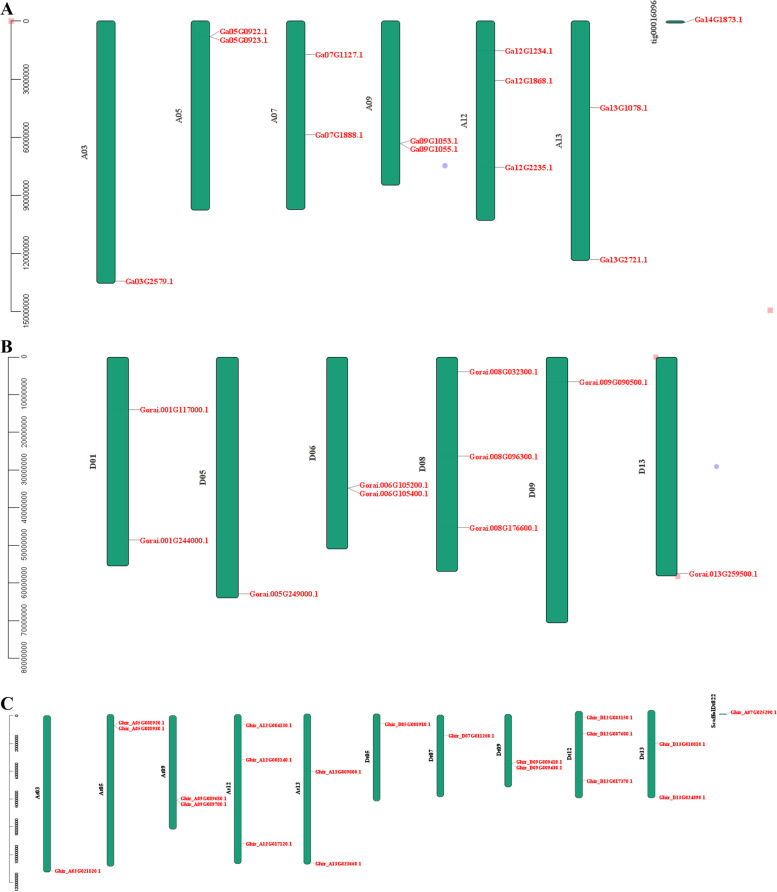
Chromosome localization of the three cotton species represented in megabases scale. The gene ID on the right side of each chromosome shows the corresponding location of ASN genes. **A** *Gossypium arboreum* (A-subgenome), (**B**) *Gossypium raimondii* (D-sub genome), and (**C**) *Gossypium hirsutum* (At-Dt sub-genome)

**Fig. 4:**
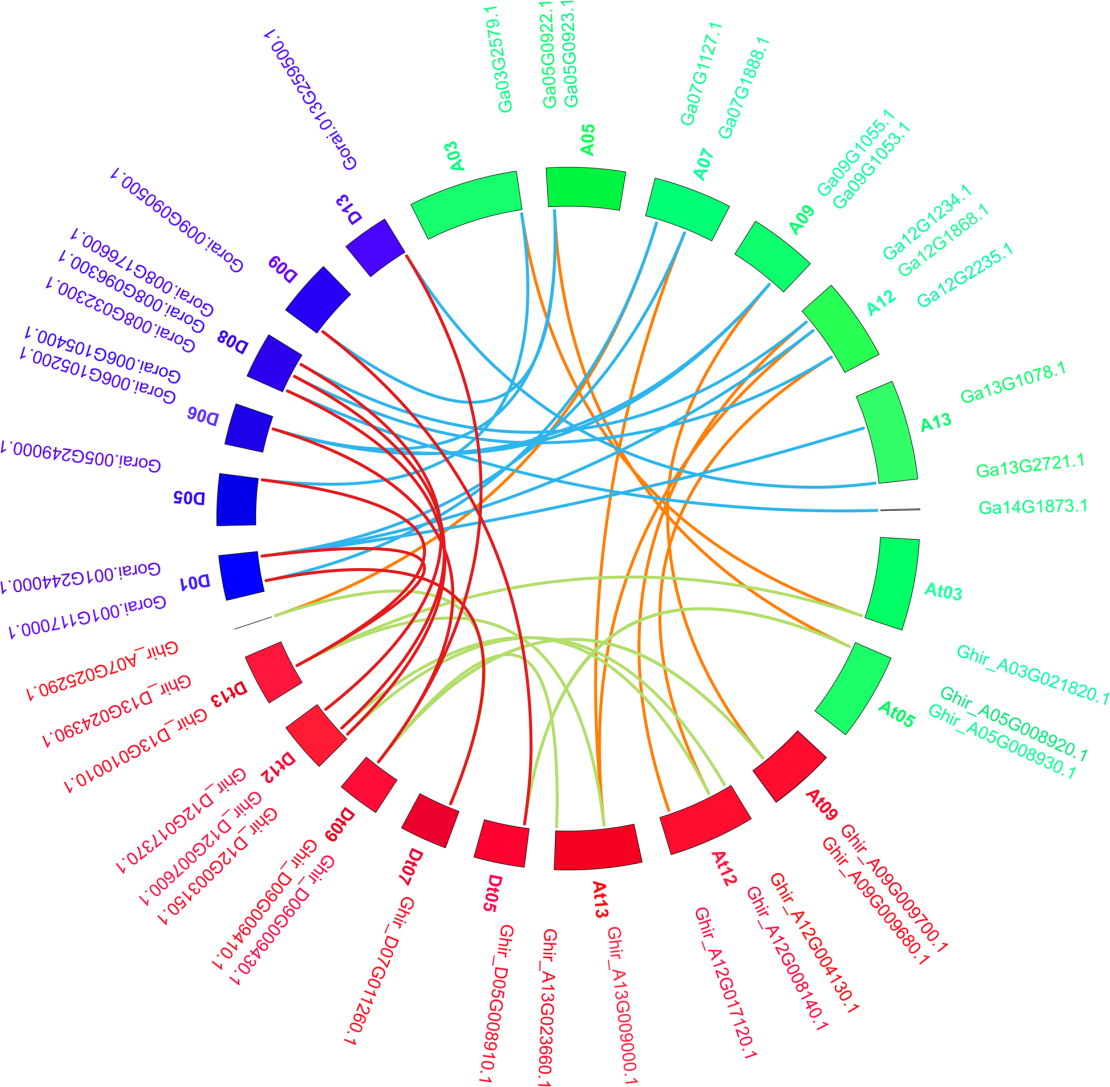
The collinearity analysis of the homologous gene pairs from three cotton species. The lines showed by different colors indicate the syntenic region around the ASN genes

The physical map of ASN members was drawn to understand the location of ASNs on chromosomes (Fig. 3). All 42 ASN genes were localized on their respective chromosomes, however, one each in *G. hirsutum* and *G. arboreum* was positioned scaffolds (Fig. 3). In *G. arboreum* (A-genome) (Fig. 3A), the highest number of ASN genes were three on chr12 followed by chr13, chr09, chr07, and chr05 each with two genes, while chr03 have only one ASN gene. No ASN member were recognized on chr01, chr02, chr04, chr06, chr08, chr10, and chr11 (Fig. 3A). In *G. raimondii* (D genome), three genes were mapped on chr08 followed by two genes each on chr06 and chr01, while, chr05, chr09, and chr13 consisted of only one ASN gene. However, no ASN members were identified in chr02, chr03, chr04, chr07, chr10, chr11, and chr12 (Fig. 3B). In *G. hirsutum* (AtDt genome) (Fig. 3C), there were no ASN genes in At01, At02, At04, At06, At07, At08, At10, At11, Dt01, Dt02, Dt03, Dt04, Dt06, Dt08, Dt10, and Dt11 chromosomes. The At subgenome consisted of ten ASN genes, which was higher than the Dt subgenome (09 genes) (Fig. 3C). Except chromosome 5, most of the homologous chromosomes of A and D had a similar number of ASN genes (Fig. 3C). The highest number of ASN on a chromosome was three on At12 and Dt12 followed by two genes in At13, Dt13, At09, Dt09, and At05. Chromosomes At03, Dt05, and Dt07 had only one ASN gene (Fig. 3C). The collinearity analysis of the ASN genes in three cotton species is represented in Fig. 4. The result showed pair-wise collinearity of the chromosomes comprising ASN genes. Besides the identification of ASN genes in A/D or At/Dt subgenome, it also demonstrated that some ASN genes were exclusively detected in the D genome. As their counterparts in the Dt subgenome of *G. hirsutum* were lost (Fig. 4).

**Fig. 5:**
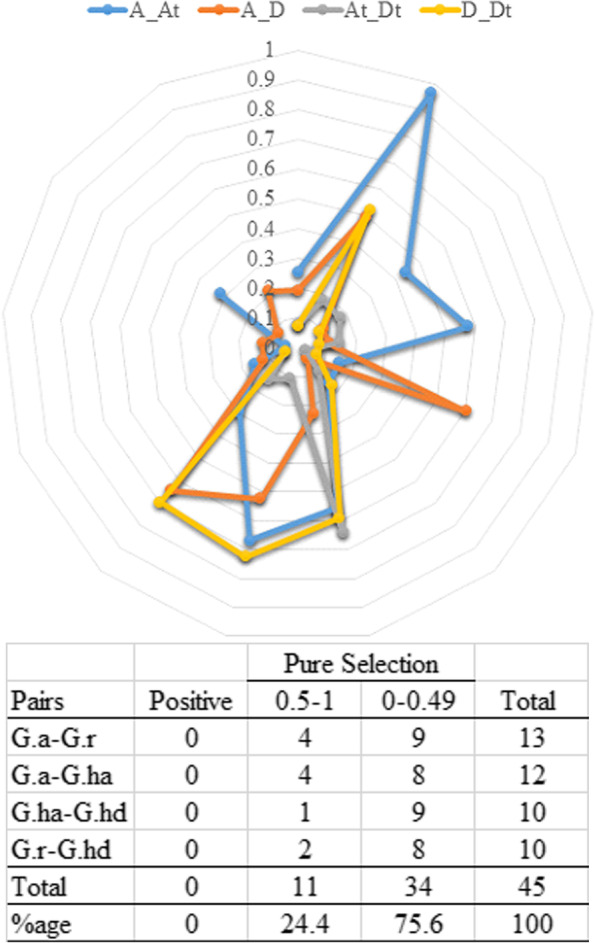
The analysis of non-synonymous (Ka) to synonymous (Ks) divergence values (A_At), (A_D), (At_Dt), and (D_Dt) are shown in a circular chart. The table shows the prediction of non-duplicated homologous gene pairs in various combinations from three cotton species. *G. arboreum* (G.a), *G. raimondii* (G.r), *G. hirsutum*; A-subgenome (G.ha), and *G. hirsutum*; D-subgenome (G.hd)

The ratio of Ka/Ks showed the stability in natural mutations, purifying selections, and positive selections on the basis of homologous genes set [48]. Therefore, Ka/Ks were analyzed to estimate the selection pressure of homologous ASN genes in cotton species. The value of Ka/Ks less than 1 showed purifying selection pressure, Ka/Ks equal to on1 is natural selection pressure, while Ka/Ks more than 1 comes in positive selection pressure. The results of Ka/Ks of homologous ASN genes in three cotton species showed a purifying selection pressure. In *G. raimondii* and *G. arboreum*, the Ka/Ks ratio of the homologous ASN genes were 0.05 to 0.64, in GhAt-Ga ranged from 0.04 to 0.97, for GhDt-Gr Ka/Ks ranged from 0.04 to 0.73, and GhAt-GhDt ranged from 0.03 to 0.64 (Fig. 5 and Table S2). The value of Ka/Ks for all the possible pairs was less than 1, suggesting that evolution was significantly slow following the purifying selection (Fig. 5 and Table S2). These results further demonstrated that the ASN genes of *G. hirsutum* derived from *G. arboreum* and *G. raimondii* have gone through negative selection and may involve a gradual elimination process during evolution.

### Identification of cis-regulatory elements in ASN family

**Fig. 6:**
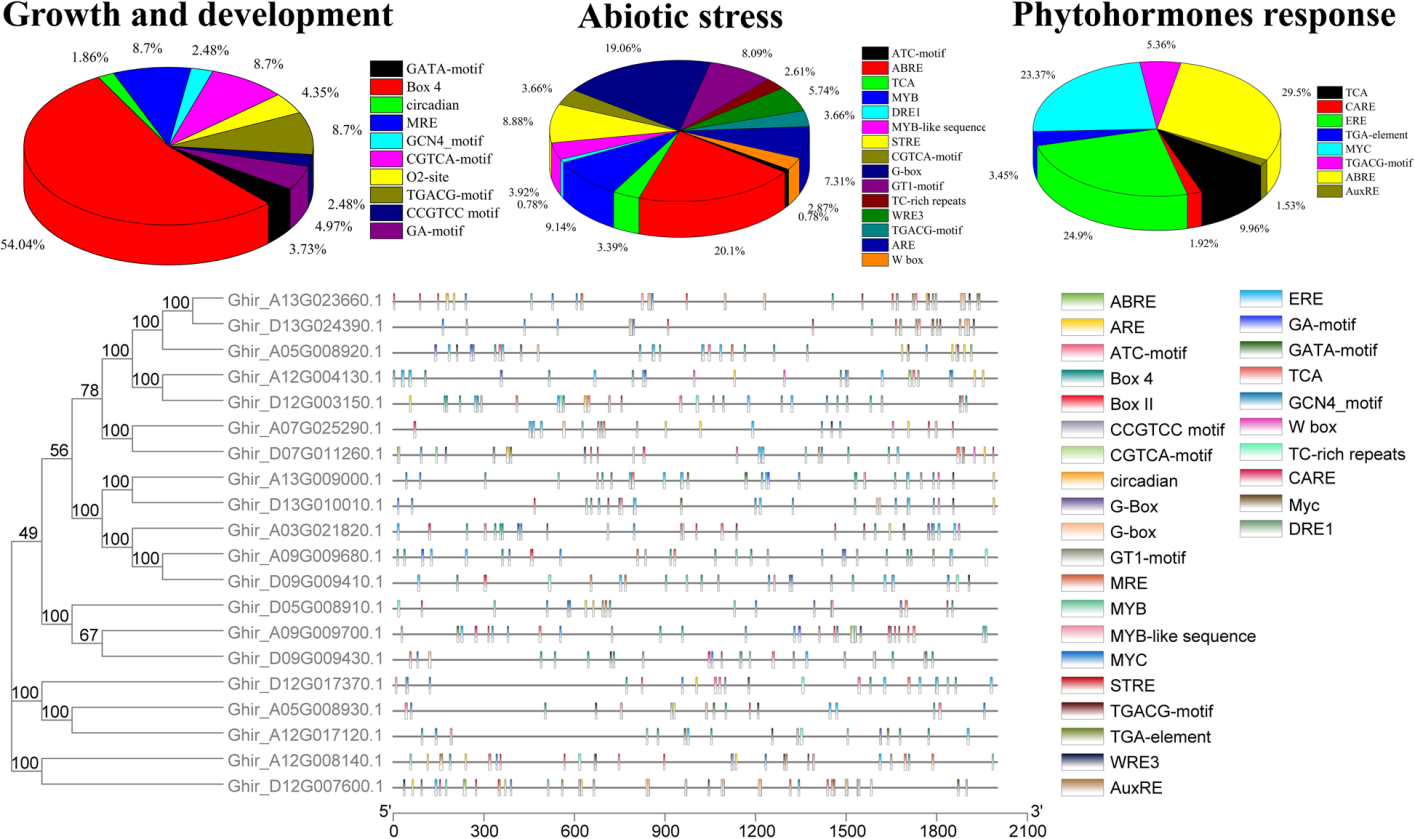
The cis-regulatory analysis of the ASN encoded genes. Different cis-elements with different functions are categorized into growth and development, abiotic stress, and hormones related cis-regulatory elements

The expression of a gene is mainly controlled by its respective promotors, followed by transcription factors (TF) in direct relation with cis-regulatory elements [49]. In the current study, we have identified cis-acting elements related to growth and development, stress, and hormones in the promotor regions of GhASN genes using the PlantCARE database. Promotor regions, consisting of 2000 bp upstream genomic DNA sequences of the transcriptional start site were examined for 20 GhASN genes. The results revealed that ASN genes may be different in function due to the availability of different cis-elements in the promoter region. The identified cis-regulatory elements were categorized into three classes such as (1) growth and development related, (2) stress-responsive, and (3) hormone-responsive (Fig. 6). The cis-elements involved in plant growth and development were located extensively in the promotor regions such as Box 4, and MRE (plant growth responsive against the light), CGTCA-motif and TGACG-motif (MejA response), GA motif (involved in light responsiveness), O2-site (responsible for zein metabolism), GATA motif (light- and nitrate-dependent control of transcription), GCN4 motif (involved in endosperm expression), CCGTCC motif (development-related element), and circadian (required for circadian control). Among the cis-elements, the largest portion (54.04%) was covered by Box 4 in the growth and development elements, followed by MRE, CGTCA, and TGACG each with 8.7% (Fig. 6 and Table S3). In the stress-responsive elements, the most abundant cis-regulatory elements include ABRE (ABA response), G-box (involved in light response), MYB (regulate auxin-related genes), STRE (defense-related elements), GT1 motif (involved in salt responsiveness), and ARE (related to anaerobic induction), WRE3 (wound response), MYB like sequence (MYB transcription factor binding site involved in drought inducibility), CGTCA-motif and TGACG-motif (MejA response), TCA (salicylic acid response element), W-box (WRKY TF involved defense response), TC rich repeats (involved in defense response), ATC motif (light-responsive), DRE1 (damage responsive elements). Among all of them, ABRE was most commonly found (20.1%), followed by G-box (19.06%), MYB (9.14%), STRE (8.88%), and GT1 motif (8.09%) (Fig. 6 and Table S3). The identified cis-regulatory elements related to plant hormones response include the ABRE (abscisic acid-responsive element), ERE (ethylene-responsive element), and MYC (abscisic acid-responsive element), TCA and TGA (salicylic acid response element), TGACG-motif (methyl jasmonate response), GARE-motif (gibberellin responsive elements), and AuxRE (auxin-responsive element). Among the hormone-responsive motifs, ABRE was the most common element (29.5%), followed by ERE (24.9%), and MYC (23.37%) (Fig. 6 and Table S3). In conclusion, ASN genes may play a key role in cotton growth and development and stress responsiveness.

### Expression profiling of ASN genes and identification of hub genes through co-expression network analysis

**Fig. 7:**
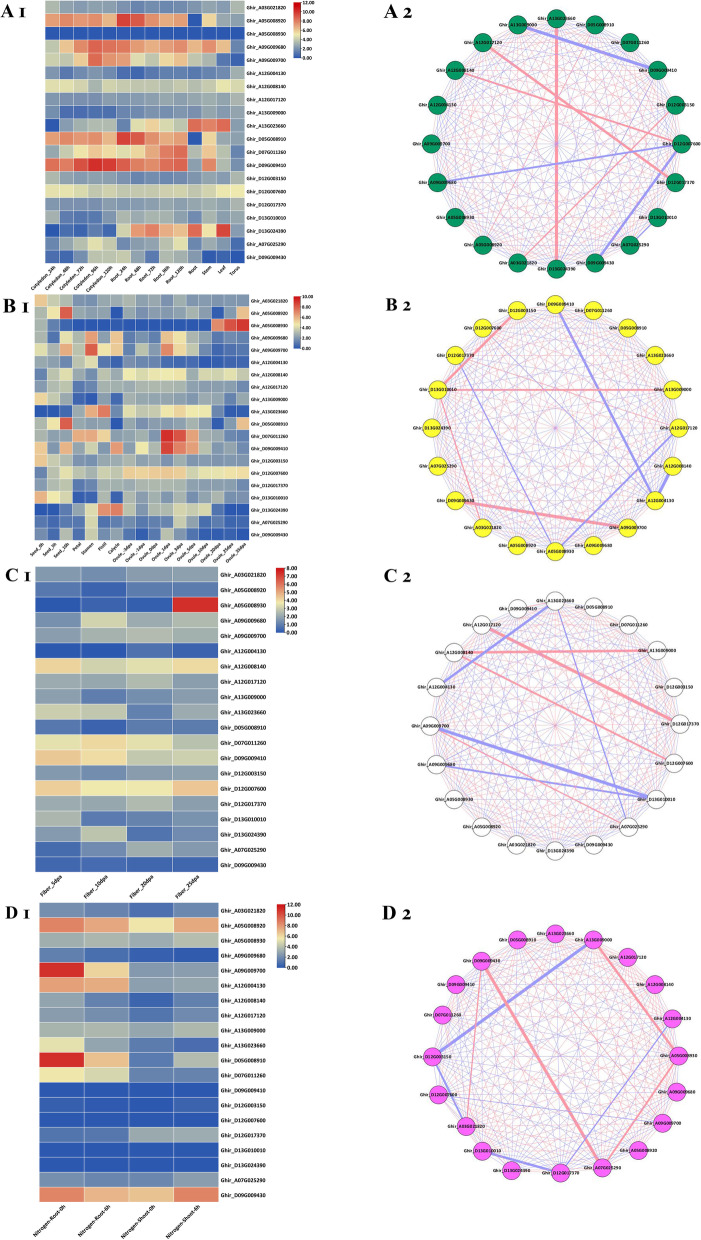
Transcriptome analysis of ASN family genes in (A1) vegetative organs, (B1) reproductive organs, (C1) fiber development, and (D1) N metabolism. The co-relation networks of 20 GhASN genes in (A2) vegetative organs, (B2) reproductive organs, (C2) fiber development, and (D2) N metabolism were analyzed on the basis of Pearson correlation of the selected ASN genes obtained from transcriptomic data. The red and blue edges showed positive and negative correlations, respectively. However, the thickness of each edge showed the strength of the correlation for each pair. In each network, the identified hub gene is represented in the center with a thick black edge

Transcriptome data were analyzed to explore the expression profiles and putative functions of ASN genes in *G. hirsutum* vegetative and reproductive organs, fiber development, and N metabolism. The results showed that *Ghir_A09G009680*, *Ghir_D09G009410*, *Ghir_D05G008910*, *Ghir_A05G008920*, *Ghir_A09G009700*, and *Ghir_A12G008140* were strongly expressed in the different vegetative organs at different time intervals like cotyledon, root, stem, and leaf, except in torus (Fig. 7A1). The other ASN genes showed slight upregulation and some were tissue-specific, while *Ghir_A05G008930* was not expressed in any vegetative organ of *G. hirsutum* (Fig. 7A1). In comparison with vegetative organs, the expression of ASN genes was less in reproductive organs, however, *Ghir_D12G007600*, *Ghir_D09G009410*, *Ghir_A12G008140*, and *Ghir_D07G011260* were the most expressed genes. *Ghir_D09G009410* and *Ghir_D07G011260* were highly expressed at early ovule development, while *Ghir_A05G008930* was only upregulated in ovule at 20 to 35 days post-anthesis (DPA) (Fig. 7B1). During fiber development, *Ghir_A12G008140*, *Ghir_D12G007600*, and *Ghir_D07G011260* were upregulated from 5 to 25 DPA, however, *Ghir_D09G009410* was upregulated at early fiber development (5–10 DPA) and *Ghir_A05G008930* was upregulated at late fiber (25 DPA) development stage, implying that they play roles in fiber development (Fig. 7C1). The ASN genes showed a high response to short-term N starvation and resupply, implying their roles in N metabolism. The results indicated that the ASN genes were mostly expressed in the roots at 0 and 6 h N-resupply (Fig. 7D1), however, *Ghir_A05G008920* and *Ghir_D09G009430* were upregulated in both roots and shoot at 0 and 6 h N-resupply (Fig. 7D1). *Ghir_A09G009700*, *Ghir_A12G004130*, *Ghir_D05G008910*, and *Ghir_D07G011260* were only expressed in root and not in the shoot, indicating their role in the root in response to N starvation and resupply (Fig. 7D1). Based on the expression pattern of RNA-Seq data, ASN genes in *G. hirsutum* are involved in vegetative and reproductive growth, fiber development, and N metabolism.

For identification of the hub genes in vegetative and reproductive organs, fiber development as well as in N metabolism, a co-expression network analysis was performed based on the correlation coefficient of their respective expression data (Fig. 7A2-D2). The co-expression network analysis of the ASN genes in vegetative and reproductive organs, fiber development, and N metabolism showed a positive and negative correlation. In vegetative organs, 91 gene pairs were positive and 99 gene pairs were negatively correlated (Fig. 7A2 and Table S4). In case of reproductive organs, 94 gene pairs had positive and 96 gene pairs had negative correlation (Fig. 7B2 and Table S4). In fiber development, 93 gene pairs had positive and 97 gene pairs had negative correlation (Fig. 7C2 and Table S4). The highest number of positive gene pairs of 108 was found in N metabolism with 82 negatively correlated gene pairs (Fig. 7D2 and Table S4). After removing the low correlation pairs, there were 36 (28 positive and 8 negative) correlation pairs in vegetative organs, 28 (24 positive and 4 negative) in reproductive organs, 127 (56 positive and 71 negative) in fiber development, and 140 (80 positive and 60 negative) in N metabolism. Based on the high number of positive correlation and high expression, we identify one hub gene each in vegetative (*Ghir_A12G008140*) and reproductive organs (*Ghir_D13G010010*), fiber development (*Ghir_A12G008140*), and N metabolism (*Ghir_D09G009430*). Moreover, the co-expression network reveals that ASN genes play a key role in N metabolism > vegetative growth > reproductive growth and > fiber development.

## Discussion

Asparagine synthetase (ASN) is an aminotransferase determined by a small and simple gene family that is broadly found in plants [50]. ASN uses ammonium and glutamine to form asparagine, which plays a key role in N transport and metabolism in plants [4, 50]. Asparagine is well known for N transport and storage that is produced from glutamine amide group and aspartate in the presence of ASN [33]. The asparagine transportation permits the required amide to be transported to various parts of the plants [51]. Moreover, many researchers have confirmed the role of ASN in N transport and metabolism [4, 23, 33, 52]. Conversely, such type of study on cotton ASNs is still to be elucidated. The recent development in the cotton sequenced genome has made it possible to extensively study the potential functions of ASN genes in cotton. In the current study, we identified the ASN genes in *G. hirsutum, G. arboreum,* and *G. raimondii* alongside *A. thaliana, O. sativa,* and *T. cacao*. The major focus was on *G. hirsutum* with the aim to widely understand the ASN gene family functions, evolution, selection pressure, family expansion, and expression profiling in cotton vegetative and reproductive organs, fiber development, and N metabolism. The current study will provide a foundation and important information for future investigations and functions of ASN genes in cotton.

The aim of the current study was to characterize the ASN gene family and its function in the growth, development, stress response, and N metabolism in cotton. Forty-three ASN protein sequences (20 from *G. hirsutum*, 13 from *G. arboreum*, and 10 from *G. raimondii*) were retrieved from cottonFGD that were further divided into three subgroups through phylogenetic analysis (Fig. 1). Interestingly, all three subgroups consisted of ASN members from all the used species suggesting their evolutionary relationship from common ancestors. Moreover, group I and III shared the maximum number of ASNs (90% from *G. hirsutum*) suggesting the significance and involvement in a large-scale expansion among the selected plant species. There was a big variation in the distribution of ASNs among the selected plant species, such as 10, 8, and 10 genes in *A. thaliana*, *T. cacao*, and *O. sativa* as compared to 20 genes in *G. hirsutum* demonstrating the conservation of ASN genes during evolution and gone through a huge scale expansion in higher plants. Moreover, the ASN genes of cotton and cacao represented a close relationship in the phylogenetic analysis firming the previous results that cotton and cacao shared a common precursor [47].

The transcription factors and cis-elements regulate the transcription process followed by the expression of respective genes. In the current study, various cis-regulatory elements related to cotton growth and development, stress, and hormone-responsive were estimated in the promotor regions of GhASNs (Fig. 6). The identified cis-regulatory elements mainly involved in growth and development were Box-4, MRE, CGTCA, and TGACG, while ABRE, G-box, MYB, STRE, GT1, and GCN4 motifs were mainly involved in abiotic stress responses (Fig. 6 and Table S3). In line with the current study, GCN4 motif was also noted in ASN1 of wheat [53]. In the phytohormones response category, ABRE, ERE, and MYC were the most common elements. Consequently, cotton ASN genes are responsible to play a key role in the growth and development, and mitigation of various environmental stresses. The results of earlier studies are in support of this hypothesis that ASN is involved in plant growth and development, stress, and hormones responses. For example, the asn2 mutant showed a poor tolerance against salinity stress in Arabidopsis and the ASN homologous gene ASN1 has been responsible for ammonium recycling against pathogen infection [54, 55]. However, the functional roles of ASNs in various environmental stresses need further investigation. Furthermore, the functional protein domain analysis demonstrated the family typically encoded ASN domain, and the expression profiling of ASNs was different in different plant vegetative and reproductive organs, fiber development as well as N metabolism, and interestingly it was in line with the ASN expression in other studied species [24, 56]. The main output of the expression profiling is the involvement of ASN genes in N metabolism in cotton.

The promoter regions of genes have several but precise cis-elements that regulate the expression of the gene in a direct combination with the transcription factors [49]. The cis-elements and various stress-responsive genes showed a positive linear correlation with each other [57]. The expression of a gene is a significant source that provides valuable indications about the function of a gene. In the current study, the transcript level of ASNs was the highest in the vegetative and reproductive organs such as cotyledon, root, stem, leaf, pistil, petal, stamen, calycle, ovule, and seed, which is in line with TaASN1 and TaASN2 expression in wheat as well as AtASN1 in Arabidopsis [6, 53]. It’s been hypothesized that the higher expression of ASNs in vegetative organs is linked with the accumulation of free amino acids and protein synthesis, probably for efficient N uptake and metabolism in cotton [1, 4], germination, and reproductive organs development [6]. Previously, it was observed that exogenous application of ABA-induced the expression of TaASN1 in wheat [58], and we have found that the ABA-responsive element (ABRE) was the most abundant among cis-regulatory elements of ASNs (Fig. 6 and Table S3). In addition, ABA is also involved in seed germination and seed development at the reproductive stage of plants [58, 59]. Thus, the high expression of ASNs in the reproductive organs may be essential for reproductive organs development and later in seed germination. However, the interaction between ASNs and ABA and their role in germination and the development of reproductive organs needs detailed investigations.

The TaASN1 expression in high-yielding wheat was higher as compared to low-yielding wheat, especially at tillering stage suggesting the role of ASN in N remobilization [60]. Therefore, ASN genes could be used as a potential marker to increase N metabolism and use efficiency [4, 60]. In our earlier study, a strong correlation between N utilization and expression of ASN genes was found in cotton [4]. Moreover, the overexpression of AtASN1 increased soluble proteins in Arabidopsis seeds, and the mutant plant showed more tolerance to N deficiency than control [61]. The asparagine level in rice drastically reduced in Osasn1 mutant leading to suppression of tiller buds growth [62]. The expression profiling of the current study suggests that ASN genes are involved in N metabolism and might be an efficient utilization of N in cotton. Finally, the results of the current study provide the basis for further molecular investigation of functional involvement of ASN genes in cotton growth and development and N metabolism.

## Conclusions

The current study provided an extensive analysis of the ASN gene family for the first time in the sequenced genome of three cotton species. In this study, twenty ASN genes were recognized in *G. hirsutum* that were unequally mapped on ten chromosomes and were divided into three subgroups by phylogenetic analysis. It was found that all ASN genes have conserved ASN and GATase domains. The expression profiling of ASNs indicated that these genes have a vital function in cotton growth and development as well as in N metabolism. Furthermore, the gene co-expression network analysis identified four hub genes one each from vegetative growth (*Ghir_A12G008140*), reproductive growth (*Ghir_D13G010010*), fiber development (*Ghir_A12G008140*), and N metabolism (*Ghir_D09G009430*). Finally, the current study provides a foundation to investigate the functions of the identified hub genes in cotton growth and development and N metabolism at the molecular level.

## Supplementary Information

**Additional file 1: Table S1.** Biophysical properties of GhASN genes including locus ID, protein length, molecular weight, isoelectric point, charge, and gravity.

**Additional file 2: Figure S1.** Identification of conserved motifs of ASN genes in three *Gossypium* species.

**Additional file 3: Table S2.** Ka/Ks (non-synonymous/synonymous) values of all identified ASN gene pairs from *G. hirsutum* (Gh; At-A genome and Dt-D genome), *G. arboreum* (Ga), and *G. raimondii* (Gr).

**Additional file 4: Table S3.** Promoter analysis; cis-element predicted in GhASN genes were involved in Growth and regulation, abiotic stress responses, and phytohormones response.

**Additional file 5: Table S4.** List of source and target traits used for correlation network based on correlation coefficient.

## Data Availability

All of the data and materials supporting our research findings are contained in the methods section of the manuscript. Details are provided in the attached Additional files. The datasets generated and/or analyzed during the current study are available in the NCBI repository [PRJNA248163 and PRJNA606910]. The Genome sequence and annotation datasets that supported our findings are available in: COTTON: https://www.cottongen.org/ Other species: https://jgi.doe.gov/data-and-tools/phytozome/.

## Abbreviations

ASN: Asparagine synthetase
N: Nitrogen
GATase: Glutamine amidotransferases
Met: Methionine
Cys: Cysteine
Gly: Glycine
Ile: Isoleucine
chr: Chromosome
DPA: Days post-anthesis
Ka: the number of non-synonymous substitutions per nonsynonymous site
Ks: the number of synonymous substitutions per synonymous site
At: *Arabidopsis thaliana*
Ga: *Gossypium arboreum*
Gh: *Gossypium hirsutum*
Gr: *Gossypium raimondii*

## References

[1] Iqbal A, Qiang D, Zhun W, Xiangru W, Huiping G, Hengheng Z (2020). Growth and nitrogen metabolism are associated with nitrogen-use efficiency in cotton genotypes. Plant Physiol Biochem.

[2] Iqbal A, Dong Q, Wang X, Gui H, Zhang H, Zhang X (2020). Variations in nitrogen metabolism are closely linked with nitrogen uptake and utilization efficiency in cotton genotypes under various nitrogen supplies. Plants.

[3] Iqbal A, Qiang D, Alamzeb M, Xiangru W, Huiping G, Hengheng Z (2020). Untangling the molecular mechanisms and functions of nitrate to improve nitrogen use efficiency. J Sci Food Agric.

[4] Iqbal A, Dong Q, Wang X, Gui H, Zhang H, Zhang X (2020). Transcriptome analysis reveals differences in key genes and pathways regulating carbon and nitrogen metabolism in cotton genotypes under n starvation and resupply. Int J Mol Sci.

[5] Iqbal A, Dong Q, Wang X, Gui H, Zhang H, Zhang X (2020). High nitrogen enhance drought tolerance in cotton through antioxidant enzymatic activities, nitrogen metabolism and osmotic adjustment. Plants.

[6] Gaufichon L, Marmagne A, Belcram K, Yoneyama T, Sakakibara Y, Hase T (2017). ASN 1-encoded asparagine synthetase in floral organs contributes to nitrogen filling in Arabidopsis seeds. Plant J.

[7] Potel F, Valadier MH, Ferrario-Méry S, Grandjean O, Morin H, Gaufichon L (2009). Assimilation of excess ammonium into amino acids and nitrogen translocation in Arabidopsis thaliana–roles of glutamate synthases and carbamoylphosphate synthetase in leaves. FEBS J.

[8] Coruzzi GM. Primary N-assimilation into amino acids in Arabidopsis. Arabidopsis Book/Am Soc Plant Biol. 2003;2:e0010. 10.1199/tab.0010 PMC3243381 22303223

[9] Funayama K, Kojima S, Tabuchi-Kobayashi M, Sawa Y, Nakayama Y, Hayakawa T (2013). Cytosolic glutamine synthetase1; 2 is responsible for the primary assimilation of ammonium in rice roots. Plant Cell Physiol.

[10] Díaz-Leal JL, Gálvez-Valdivieso G, Fernández J, Pineda M, Alamillo JM (2012). Developmental effects on ureide levels are mediated by tissue-specific regulation of allantoinase in Phaseolus vulgaris L. J Exp Bot.

[11] Lomelino CL, Andring JT, McKenna R, Kilberg MS (2017). Asparagine synthetase: function, structure, and role in disease. J Biol Chem.

[12] Gaufichon L, Reisdorf-Cren M, Rothstein SJ, Chardon F, Suzuki A (2010). Biological functions of asparagine synthetase in plants. Plant Sci.

[13] Muttucumaru N, Keys AJ, Parry MA, Powers SJ, Halford NG (2014). Photosynthetic assimilation of 14 C into amino acids in potato (*Solanum tuberosum*) and asparagine in the tubers. Planta.

[14] Van Heeke G, Schuster SM (1989). Expression of human asparagine synthetase in *Escherichia coli*. J Biol Chem.

[15] Lam H-M, Peng SS, Coruzzi GM (1994). Metabolic regulation of the gene encoding glutamine-dependent asparagine synthetase in *Arabidopsis thaliana*. Plant Physiol.

[16] Davies KM, King GA (1993). Isolation and characterization of a cDNA clone for a harvest-induced asparagine synthetase from *Asparagus officinalis* L. Plant Physiol.

[17] Mei B, Zalkin H (1989). A cysteine-histidine-aspartate catalytic triad is involved in glutamine amide transfer function in purF-type glutamine amidotransferases. J Biol Chem.

[18] Mäntsälä P, Zalkin H (1992). Cloning and sequence of *Bacillus subtilis* purA and guaA, involved in the conversion of IMP to AMP and GMP. J Bacteriol.

[19] Shi L, Twary SN, Yoshioka H, Gregerson RG, Miller SS, Samac DA (1997). Nitrogen assimilation in alfalfa: isolation and characterization of an asparagine synthetase gene showing enhanced expression in root nodules and dark-adapted leaves. Plant Cell.

[20] Lam HM, Hsieh MH, Coruzzi G (1998). Reciprocal regulation of distinct asparagine synthetase genes by light and metabolites in *Arabidopsis thaliana*. Plant J.

[21] Gálvez-Valdivieso G, Alamillo JM, Fernández J, Pineda M (2013). Molecular characterization of PVAS3: an asparagine synthetase gene from common bean prevailing in developing organs. J Plant Physiol.

[22] Herrera-Rodríguez MB, Carrasco-Ballesteros S, Maldonado JM, Pineda M, Aguilar M, Pérez-Vicente R (2002). Three genes showing distinct regulatory patterns encode the asparagine synthetase of sunflower (*Helianthus annuus*). New Phytol.

[23] Gaufichon L, Masclaux-Daubresse C, Tcherkez G, Reisdorf-Cren M, Sakakibara Y, Hase T (2013). *Arabidopsis thaliana* ASN2 encoding asparagine synthetase is involved in the control of nitrogen assimilation and export during vegetative growth. Plant Cell Environ.

[24] Wong H-K, Chan H-K, Coruzzi GM, Lam H-M (2004). Correlation of ASN2 gene expression with ammonium metabolism in Arabidopsis. Plant Physiol.

[25] Osuna D, Gálvez-Valdivieso G, Piedras P, Pineda M, Aguilar M. Cloning, characterization and mRNA expression analysis of PVAS1, a type I asparagine synthetase gene from *Phaseolus vulgaris*. Planta. 2001;213(3):402–10. 10.1007/s004250000513 11506363

[26] Antunes F, Aguilar M, Pineda M, Sodek L (2008). Nitrogen stress and the expression of asparagine synthetase in roots and nodules of soybean (*Glycine max*). Physiol Plant.

[27] Hayashi H, Chino M (1990). Chemical composition of phloem sap from the uppermost internode of the rice plant. Plant Cell Physiol.

[28] Dembinski E, Bany S (1991). The amino acid pool of high and low protein rye inbred lines (*Secale cereale* L.). J Plant Physiol.

[29] Dembinski E, Bany S, Raczynska-Bojanowska K. Asparagine and glutamine in the leaves of high and low protein maize. Acta Physiol Plant. 1995;17(4):361–65.

[30] Lohaus G, Büker M, Hußmann M, Soave C, Heldt H-W (1998). Transport of amino acids with special emphasis on the synthesis and transport of asparagine in the Illinois low protein and Illinois high protein strains of maize. Planta.

[31] Wan T-F, Shao G-H, Shan X-C, Zeng N-Y, Lam H-M (2006). Correlation between AS1 gene expression and seed protein contents in different soybean (*Glycine max* [L.] Merr.) cultivars. Plant Biol.

[32] Ohashi M, Ishiyama K, Kojima S, Konishi N, Nakano K, Kanno K (2015). Asparagine synthetase1, but not asparagine synthetase2, is responsible for the biosynthesis of asparagine following the supply of ammonium to rice roots. Plant Cell Physiol.

[33] Herrera-Rodríguez MB, Maldonado JM, Pérez-Vicente R (2006). Role of asparagine and asparagine synthetase genes in sunflower (*Helianthus annuus*) germination and natural senescence. J Plant Physiol.

[34] Gregersen PL, Culetic A, Boschian L, Krupinska K (2013). Plant senescence and crop productivity. Plant Mol Biol.

[35] Fischer AM (2012). The complex regulation of senescence. Crit Rev Plant Sci.

[36] Whitelaw C, Barbazuk W, Pertea G, Chan A, Cheung F, Lee Y (2003). Enrichment of gene-coding sequences in maize by genome filtration. Science.

[37] Zhu T, Liang C, Meng Z, Sun G, Meng Z, Guo S (2017). CottonFGD: an integrated functional genomics database for cotton. BMC Plant Biol.

[38] Mistry J, Finn R (2007). A domain-centric method for analyzing proteins and proteomes. Compar Genom.

[39] Wu X, Song C, Wang B, Cheng J (2002). Hidden Markov model used in protein sequence analysis. Sheng wu yi xue gong cheng xue za zhi= J Biomed Engineering= Shengwu yixue gongchengxue zazhi.

[40] Jeanmougin F, Thompson JD, Gouy M, Higgins DG, Gibson TJ (1998). Multiple sequence alignment with Clustal X. Trends Biochem Sci.

[41] Kumar S, Stecher G, Tamura K (2016). MEGA7: molecular evolutionary genetics analysis version 7.0 for bigger datasets. Mol Biol Evol.

[42] Edgar RC (2004). MUSCLE: a multiple sequence alignment method with reduced time and space complexity. BMC Bioinform.

[43] Tamura K, Peterson D, Peterson N, Stecher G, Nei M, Kumar S (2011). MEGA5: molecular evolutionary genetics analysis using maximum likelihood, evolutionary distance, and maximum parsimony methods. Mol Biol Evol.

[44] Chen C, Chen H, He Y, Xia R. TBtools, a toolkit for biologists integrating various biological data handling tools with a user-friendly interface. BioRxiv. 2018;289660.

[45] Hu Y, Chen J, Fang L, Zhang Z, Ma W, Niu Y (2019). *Gossypium barbadense* and *Gossypium hirsutum* genomes provide insights into the origin and evolution of allotetraploid cotton. Nat Genet.

[46] Asif I, Dong Q, Wang X, Gui H, Zhang H, Pang N (2021). Genotypic variation in root morphology, cotton subtending leaf physiology and fiber quality against nitrogen. J Cotton Res.

[47] Li F, Fan G, Wang K, Sun F, Yuan Y, Song G (2014). Genome sequence of the cultivated cotton *Gossypium arboreum*. Nat Genet.

[48] Hurst LD (2002). The Ka/Ks ratio: diagnosing the form of sequence evolution. Trends Genet: TIG.

[49] Higo K, Ugawa Y, Iwamoto M, Korenaga T (1999). Plant cis-acting regulatory DNA elements (PLACE) database: 1999. Nucleic Acids Res.

[50] Qu C, Hao B, Xu X, Wang Y, Yang C, Xu Z (2019). Functional research on three presumed asparagine synthetase family members in poplar. Genes.

[51] Waterhouse RN, Smyth AJ, Massonneau A, Prosser IM, Clarkson DT (1996). Molecular cloning and characterisation of asparagine synthetase from *Lotus japonicus*: dynamics of asparagine synthesis in N-sufficient conditions. Plant Mol Biol.

[52] Martínez-Andújar C, Ghanem ME, Albacete A, Pérez-Alfocea F (2013). Response to nitrate/ammonium nutrition of tomato (*Solanum lycopersicum* L.) plants overexpressing a prokaryotic NH_4_^+^-dependent asparagine synthetase. J Plant Physiol.

[53] Gao R, Curtis TY, Powers SJ, Xu H, Huang J, Halford NG (2016). Food safety: structure and expression of the asparagine synthetase gene family of wheat. J Cereal Sci.

[54] Maaroufi-Dguimi H, Debouba M, Gaufichon L, Clément G, Gouia H, Hajjaji A (2011). An Arabidopsis mutant disrupted in ASN2 encoding asparagine synthetase 2 exhibits low salt stress tolerance. Plant Physiol Biochem.

[55] Olea F, Pérez-García A, Cantón FR, Rivera ME, Cañas R, Ávila C (2004). Up-regulation and localization of asparagine synthetase in tomato leaves infected by the bacterial pathogen *pseudomonas syringae*. Plant Cell Physiol.

[56] Herrera-Rodríguez MB, Maldonado JM, Pérez-Vicente R (2004). Light and metabolic regulation of HAS1, HAS1. 1 and HAS2, three asparagine synthetase genes in *Helianthus annuus*. Plant Physiol Biochem.

[57] Tran LSP, Nakashima K, Sakuma Y, Osakabe Y, Qin F, Simpson SD (2007). Co-expression of the stress-inducible zinc finger homeodomain ZFHD1 and NAC transcription factors enhances expression of the ERD1 gene in Arabidopsis. Plant J.

[58] Wang H, Liu D, Sun J, Zhang A (2005). Asparagine synthetase gene TaASN1 from wheat is up-regulated by salt stress, osmotic stress and ABA. J Plant Physiol.

[59] Huang Y, Sun M-M, Ye Q, Wu X-Q, Wu W-H, Chen Y-F (2017). Abscisic acid modulates seed germination via ABA insensitive5-mediated phosphate1. Plant Physiol.

[60] Tian H, Fu J, Drijber RA, Gao Y (2015). Expression patterns of five genes involved in nitrogen metabolism in two winter wheat (*Triticum aestivum* L.) genotypes with high and low nitrogen utilization efficiencies. J Cereal Sci.

[61] Lam H-M, Wong P, Chan H-K, Yam K-M, Chen L, Chow C-M (2003). Overexpression of the ASN1 gene enhances nitrogen status in seeds of Arabidopsis. Plant Physiol.

[62] Luo L, Qin R, Liu T, Yu M, Yang T, Xu G (2019). OsASN1 plays a critical role in asparagine-dependent rice development. Int J Mol Sci.

