# Supplementary material for: Genome-wide expression analysis reveals involvement of asparagine synthetase family in cotton development and nitrogen metabolism
Source: BMC Plant Biol. 2022 Mar 16;22:122. doi: 10.1186/s12870-022-03454-7 (PMC8925137; doi:10.1186/s12870-022-03454-7)
Supplement: Supplementary file 1 — Additional file 1: Table S1. Biophysical properties of GhASN genes including locus ID, protein length, molecular weight, isoelectric point, charge, and gravity. [file 12870_2022_3454_MOESM1_ESM.docx]

Additional file 1: Table S1. Biophysical properties of GhASN genes including locus ID, protein length, molecular weight, isoelectric point, charge, and gravity.

| **Gene ID** | **Protein length (aa)** | **Molecular weight (kDa)** | **Charge** | **Isoelectric point** | **Grand average of hydropathy** |
| --- | --- | --- | --- | --- | --- |
| Ghir_D12G017370 | 424 | 47.79 | 2.50 | 7.008 | -0.387 |
| Ghir_A12G008140 | 568 | 64.12 | 4.50 | 6.920 | -0.336 |
| Ghir_D12G007600 | 568 | 64.07 | 3.00 | 6.788 | -0.325 |
| Ghir_A07G025290 | 584 | 66.03 | 4.50 | 6.818 | -0.320 |
| Ghir_D07G011260 | 584 | 65.86 | 1.50 | 6.608 | -0.312 |
| Ghir_D09G009410 | 583 | 65.60 | 0.50 | 6.546 | -0.296 |
| Ghir_A09G009680 | 583 | 65.56 | 0.50 | 6.546 | -0.290 |
| Ghir_A09G009700 | 554 | 62.51 | -2.50 | 6.331 | -0.281 |
| Ghir_D13G024390 | 498 | 56.16 | 1.00 | 6.584 | -0.277 |
| Ghir_A05G008930 | 561 | 63.11 | 7.00 | 7.095 | -0.268 |
| Ghir_A05G008920 | 585 | 65.73 | 4.00 | 6.796 | -0.262 |
| Ghir_D05G008910 | 585 | 65.89 | 2.00 | 6.662 | -0.260 |
| Ghir_D09G009430 | 544 | 61.39 | -6.00 | 6.036 | -0.257 |
| Ghir_A13G023660 | 362 | 40.35 | 1.00 | 6.630 | -0.251 |
| Ghir_A12G017120 | 546 | 61.52 | -4.00 | 5.971 | -0.247 |
| Ghir_D13G010010 | 589 | 64.35 | -1.50 | 6.352 | -0.099 |
| Ghir_A13G009000 | 578 | 63.12 | 4.00 | 7.003 | -0.068 |
| Ghir_A12G004130 | 559 | 61.01 | 4.50 | 7.119 | -0.050 |
| Ghir_A03G021820 | 582 | 63.74 | 2.50 | 6.825 | -0.025 |
| Ghir_D12G003150 | 586 | 63.75 | 0.00 | 6.513 | -0.020 |
