# Supplementary material for: Genome-wide expression analysis reveals involvement of asparagine synthetase family in cotton development and nitrogen metabolism
Source: BMC Plant Biol. 2022 Mar 16;22:122. doi: 10.1186/s12870-022-03454-7 (PMC8925137; doi:10.1186/s12870-022-03454-7)

Additional file 2: Figure S1. Identification of conserved motifs of ASN genes in three *Gossypium* species.


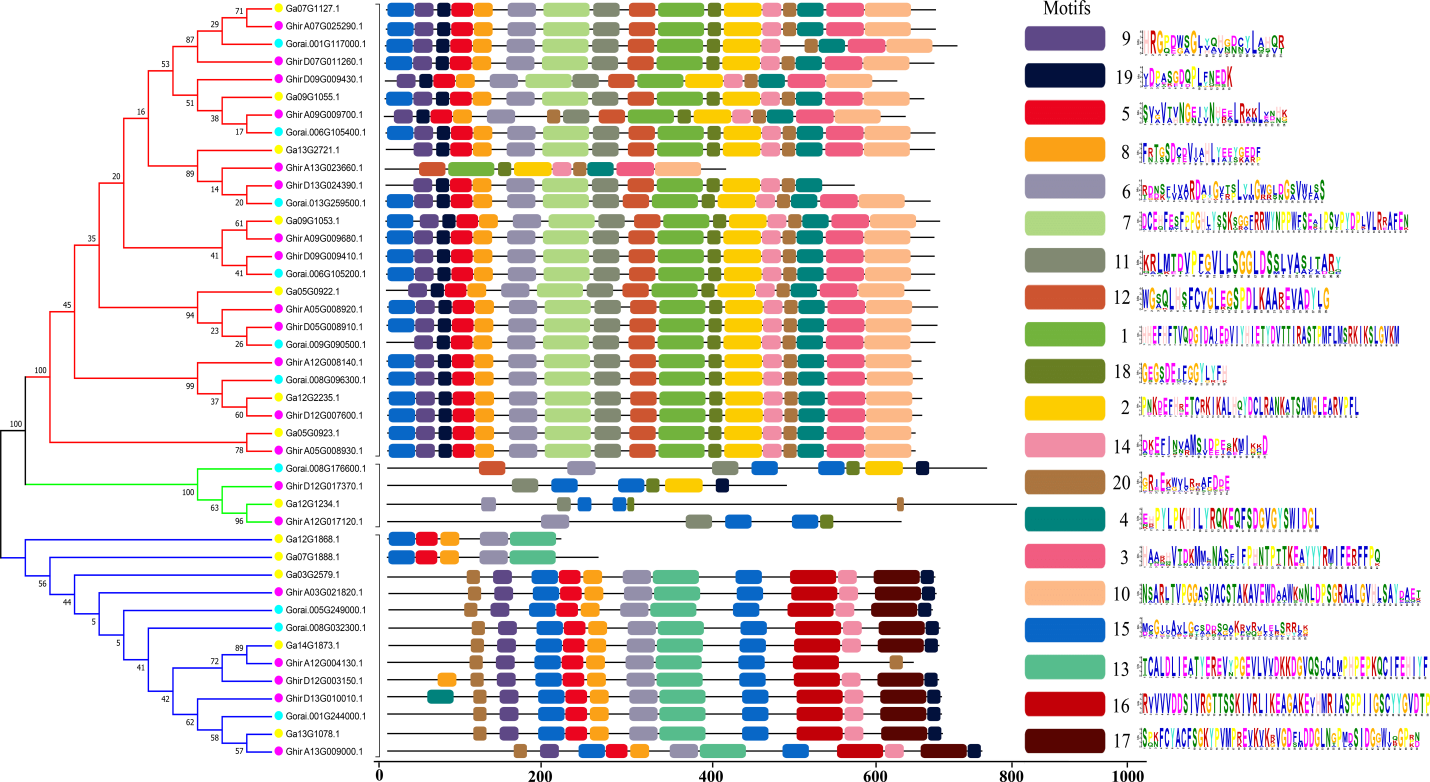

Supplement: Supplementary file 2 — Additional file 2: Figure S1. Identification of conserved motifs of ASN genes in three Gossypium species. [file 12870_2022_3454_MOESM2_ESM.docx]
