# Supplementary material for: Genome-wide expression analysis reveals involvement of asparagine synthetase family in cotton development and nitrogen metabolism
Source: BMC Plant Biol. 2022 Mar 16;22:122. doi: 10.1186/s12870-022-03454-7 (PMC8925137; doi:10.1186/s12870-022-03454-7)
Supplement: Supplementary file 3 — Additional file 3: Table S2. Ka/Ks (non-synonymous/synonymous) values of all identified ASN gene pairs from G. hirsutum (Gh; At-A genome and Dt-D genome), G. arboreum (Ga), and G. raimondii (Gr). [file 12870_2022_3454_MOESM3_ESM.docx]

Additional file 3: Table S2. Ka/Ks (non-synonymous/synonymous) values of all identified ASN gene pairs from *G. hirsutum* (Gh; At-A genome and Dt-D genome), *G. arboreum* (Ga), and *G. raimondii* (Gr).

| **Ga-Seq** | **GhAt-Seq** | **Ka** | **Ks** | **Ka/Ks** | **Effective Len** | **Average S-sites** | **Average N-sites** |
| --- | --- | --- | --- | --- | --- | --- | --- |
| Ga03G2579.1 | Ghir_A03G021820.1 | 0.003024 | 0.011986 | 0.252282 | 1746 | 420.5 | 1325.5 |
| Ga05G0922.1 | Ghir_A05G008920.1 | 0.027493 | 0.028414 | 0.967571 | 1728 | 397.5 | 1330.5 |
| Ga05G0923.1 | Ghir_A05G008930.1 | 0.003863 | 0 | 0 | 1683 | 385.3333 | 1297.667 |
| Ga07G1127.1 | Ghir_A07G025290.1 | 0.002223 | 0.005014 | 0.443322 | 1752 | 400.25 | 1351.75 |
| Ga07G1888.1 | Ghir_A13G009000.1 | 0.051596 | 0.089165 | 0.578662 | 672 | 160.5833 | 511.4167 |
| Ga09G1053.1 | Ghir_A09G009680.1 | 0.00149 | 0.009942 | 0.149824 | 1749 | 405 | 1344 |
| Ga09G1055.1 | Ghir_D09G009410.1 | 0.025325 | 0.158335 | 0.159945 | 1719 | 400.5 | 1318.5 |
| Ga12G1234.1 | Ghir_A12G017120.1 | 0.065939 | 0.118703 | 0.5555 | 1638 | 377.25 | 1260.75 |
| Ga12G1868.1 | Ghir_A13G009000.1 | 0.061004 | 0.091262 | 0.668453 | 552 | 133.8333 | 418.1667 |
| Ga12G2235.1 | Ghir_A12G008140.1 | 0.001521 | 0.005172 | 0.294117 | 1704 | 388 | 1316 |
| Ga13G1078.1 | Ghir_D13G010010.1 | 0.00597 | 0.038938 | 0.153325 | 1767 | 421.6667 | 1345.333 |
| Ga13G2721.1 | Ghir_D09G009410.1 | 0.035662 | 0.865336 | 0.041212 | 1746 | 403.6667 | 1342.333 |
| Ga14G1873.1 | Ghir_D12G003150.1 | 0.012146 | 0.038439 | 0.31598 | 1755 | 427 | 1328 |
| **Ga-Seq** | **Gr-Seq** | **Ka** | **Ks** | **Ka/Ks** | **Effective Len** | **Average S-sites** | **Average N-sites** |
| Ga03G2579.1 | Gorai.005G249000.1 | 0.01103 | 0.057899 | 0.190511 | 1746 | 421.75 | 1324.25 |
| Ga05G0922.1 | Gorai.009G090500.1 | 0.035257 | 0.071206 | 0.495145 | 1710 | 391.3333 | 1318.667 |
| Ga05G0923.1 | Gorai.006G105400.1 | 0.059649 | 0.567398 | 0.105127 | 1680 | 389.4167 | 1290.583 |
| Ga07G1127.1 | Gorai.001G117000.1 | 0.006689 | 0.062455 | 0.107102 | 1752 | 400.5 | 1351.5 |
| Ga07G1888.1 | Gorai.001G244000.1 | 0.053748 | 0.088871 | 0.60479 | 672 | 161.0833 | 510.9167 |
| Ga09G1053.1 | Gorai.006G105200.1 | 0.002236 | 0.045806 | 0.048806 | 1749 | 405.0833 | 1343.917 |
| Ga09G1055.1 | Gorai.006G105400.1 | 0.01676 | 0.073623 | 0.227644 | 1719 | 401.6667 | 1317.333 |
| Ga12G1234.1 | Gorai.008G176600.1 | 0.129933 | 0.247346 | 0.525309 | 1911 | 437.8333 | 1473.167 |
| Ga12G1868.1 | Gorai.001G244000.1 | 0.063682 | 0.099351 | 0.640979 | 552 | 134.3333 | 417.6667 |
| Ga12G2235.1 | Gorai.008G096300.1 | 0.004574 | 0.036955 | 0.123774 | 1704 | 388.25 | 1315.75 |
| Ga13G1078.1 | Gorai.001G244000.1 | 0.005223 | 0.043897 | 0.118988 | 1767 | 422.1667 | 1344.833 |
| Ga13G2721.1 | Gorai.013G259500.1 | 0.005217 | 0.064835 | 0.080462 | 1749 | 402.5 | 1346.5 |
| Ga14G1873.1 | Gorai.008G032300.1 | 0.009849 | 0.045945 | 0.214357 | 1755 | 426.3333 | 1328.667 |
| **GhAt-Seq** | **GhDt-Seq** | **Ka** | **Ks** | **Ka/Ks** | **Effective Len** | **Average S-sites** | **Average N-sites** |
| Ghir_A03G021820.1 | Ghir_D13G010010.1 | 0.069605 | 0.91759 | 0.075856 | 1731 | 414.6667 | 1316.333 |
| Ghir_A05G008920.1 | Ghir_D05G008910.1 | 0.008195 | 0.045767 | 0.179064 | 1755 | 405.4167 | 1349.583 |
| Ghir_A05G008930.1 | Ghir_D05G008910.1 | 0.049942 | 0.284543 | 0.175516 | 1680 | 386.4167 | 1293.583 |
| Ghir_A07G025290.1 | Ghir_D07G011260.1 | 0.007811 | 0.055665 | 0.140324 | 1752 | 400.75 | 1351.25 |
| Ghir_A09G009680.1 | Ghir_D09G009410.1 | 0.001489 | 0.051094 | 0.029152 | 1749 | 404.9167 | 1344.083 |
| Ghir_A09G009700.1 | Ghir_D09G009410.1 | 0.015968 | 0.146243 | 0.109189 | 1659 | 382.5833 | 1276.417 |
| Ghir_A12G004130.1 | Ghir_D12G003150.1 | 0.054952 | 0.085602 | 0.641953 | 1674 | 404.8333 | 1269.167 |
| Ghir_A12G008140.1 | Ghir_D12G007600.1 | 0.004577 | 0.042302 | 0.108191 | 1704 | 389 | 1315 |
| Ghir_A13G009000.1 | Ghir_D13G010010.1 | 0.006094 | 0.042021 | 0.145035 | 1734 | 416 | 1318 |
| Ghir_A13G023660.1 | Ghir_D09G009410.1 | 0.040359 | 0.854638 | 0.047223 | 1083 | 249.5 | 833.5 |
| **Gr-Seq** | **GhDt-Seq** | **Ka** | **Ks** | **Ka/Ks** | **Effective Len** | **Average S-sites** | **Average N-sites** |
| Gorai.001G117000.1 | Ghir_D07G011260.1 | 0.001482 | 0.02022 | 0.073285 | 1752 | 401 | 1351 |
| Gorai.001G244000.1 | Ghir_D13G010010.1 | 0.003727 | 0.00714 | 0.522008 | 1767 | 422.1667 | 1344.833 |
| Gorai.005G249000.1 | Ghir_D13G010010.1 | 0.080221 | 0.871154 | 0.092086 | 1761 | 423.5833 | 1337.417 |
| Gorai.006G105200.1 | Ghir_D09G009410.1 | 7.44E-04 | 0.009942 | 0.074875 | 1749 | 405 | 1344 |
| Gorai.006G105400.1 | Ghir_D09G009410.1 | 0.00975 | 0.140065 | 0.069611 | 1749 | 407 | 1342 |
| Gorai.008G032300.1 | Ghir_D12G003150.1 | 0.004525 | 0.026152 | 0.173026 | 1758 | 428 | 1330 |
| Gorai.008G096300.1 | Ghir_D12G007600.1 | 0.003049 | 0.005156 | 0.591297 | 1704 | 389.25 | 1314.75 |
| Gorai.008G176600.1 | Ghir_D12G017370.1 | 0.00509 | 0.00702 | 0.725038 | 1272 | 286.25 | 985.75 |
| Gorai.009G090500.1 | Ghir_D05G008910.1 | 0.005251 | 0.007552 | 0.695315 | 1737 | 399.25 | 1337.75 |
| Gorai.013G259500.1 | Ghir_D09G009410.1 | 0.036416 | 0.815931 | 0.044631 | 1746 | 402.6667 | 1343.333 |
