# Supplementary material for: Genome-wide expression analysis reveals involvement of asparagine synthetase family in cotton development and nitrogen metabolism
Source: BMC Plant Biol. 2022 Mar 16;22:122. doi: 10.1186/s12870-022-03454-7 (PMC8925137; doi:10.1186/s12870-022-03454-7)
Supplement: Supplementary file 4 — Additional file 4: Table S3. Promoter analysis; cis-element predicted in GhASN genes were involved in Growth and regulation, abiotic stress responses, and phytohormones response. [file 12870_2022_3454_MOESM4_ESM.docx]

Additional 4: Table S3. Promoter analysis; cis-element predicted in GhASN genes were involved in Growth and regulation, abiotic stress responses, and phytohormones response.

| **Motif Name** | **Functions** | **No of Genes involved** | **Percentage (%)** |
| --- | --- | --- | --- |
| **Growth and Development** | |  |  |
| Box 4 | Light responsive | 87 | 54.04 |
| MRE | Light responsive | 14 | 8.70 |
| CGTCA-motif | MeJA responsiveness | 14 | 8.70 |
| TGACG-motif | Involved in auxin response | 14 | 8.70 |
| GA-motif | Involved in light responsiveness | 8 | 4.97 |
| O2-site | Regulation of zein metabolism | 7 | 4.35 |
| GATA-motif | Light- and nitrate-dependent control of transcription | 6 | 3.73 |
| GCN4_motif | Critical for endosperm expression | 4 | 2.48 |
| CCGTCC motif | Development related element | 4 | 2.48 |
| circadian | For circadian control | 3 | 1.86 |
| **Abiotic Stress** |  |  |  |
| ABRE | ABA response | 77 | 20.10 |
| G-box | Involved in light responsiveness | 73 | 19.06 |
| MYB | Plays a vital role in the regulation of auxin-regulated genes | 35 | 9.14 |
| STRE | Defense related elements | 34 | 8.88 |
| GT1-motif | Salt responsive element | 31 | 8.09 |
| ARE | Involved in anaerobic induction | 28 | 7.31 |
| WRE3 | Wound response | 22 | 5.74 |
| MYB-like sequence | MYB transcription factor binding site involved in Drought inducibility | 15 | 3.92 |
| CGTCA-motif | MejA response | 14 | 3.66 |
| TGACG-motif | Involved in auxin response | 14 | 3.66 |
| TCA | Salicylic acid responsiveness | 13 | 3.39 |
| W box | WRKY transcription factor binding site in defense responses | 11 | 2.87 |
| TC-rich repeats | Cis-acting element involved in defense and stress responsiveness | 10 | 2.61 |
| DRE1 | Damage responsive elements | 3 | 0.78 |
| ATC-motif | Light responsive | 3 | 0.78 |
| **Phytohormone Response** | |  |  |
| ABRE | Abscisic acid responsiveness | 77 | 29.50 |
| ERE | Ethylene response | 65 | 24.90 |
| MYC | Involved in abscisic acid responsiveness | 61 | 23.37 |
| TCA | Salicylic acid response element | 26 | 9.96 |
| TGACG-motif | Involved in auxin response | 14 | 5.36 |
| TGA-element | Auxin-responsive element | 9 | 3.45 |
| GARE-motif | Gibberellin-responsive element | 5 | 1.92 |
| AuxRE | Auxin responsive element | 4 | 1.53 |
