# Supplementary material for: Genome-wide expression analysis reveals involvement of asparagine synthetase family in cotton development and nitrogen metabolism
Source: BMC Plant Biol. 2022 Mar 16;22:122. doi: 10.1186/s12870-022-03454-7 (PMC8925137; doi:10.1186/s12870-022-03454-7)
Supplement: Supplementary file 5 — Additional file 5: Table S4. List of source and target traits used for correlation network based on correlation coefficient. [file 12870_2022_3454_MOESM5_ESM.docx]

Additional file 5: Table S4. List of source and target traits used for correlation network based on correlation coefficient.

| **Vegetative organs** | | |  | |  | |  | |  |
| --- | --- | --- | --- | --- | --- | --- | --- | --- | --- |
| **Source gene** | | **Target gene** | **Pcor.** | | **Source gene** | | **Target gene** | | **Pcor.** |
| Ghir_A03G021820 | | Ghir_A05G008920 | -0.03 | | Ghir_A03G021820 | | Ghir_D05G008910 | | -0.03 |
| Ghir_A03G021820 | | Ghir_A05G008930 | -0.14 | | Ghir_A05G008920 | | Ghir_D05G008910 | | 1.00 |
| Ghir_A05G008920 | | Ghir_A05G008930 | 0.69 | | Ghir_A05G008930 | | Ghir_D05G008910 | | 0.69 |
| Ghir_A03G021820 | | Ghir_A09G009680 | -0.11 | | Ghir_A09G009680 | | Ghir_D05G008910 | | 0.09 |
| Ghir_A05G008920 | | Ghir_A09G009680 | 0.09 | | Ghir_A09G009700 | | Ghir_D05G008910 | | 0.05 |
| Ghir_A05G008930 | | Ghir_A09G009680 | 0.00 | | Ghir_A12G004130 | | Ghir_D05G008910 | | -0.19 |
| Ghir_A03G021820 | | Ghir_A09G009700 | 0.07 | | Ghir_A12G008140 | | Ghir_D05G008910 | | 0.03 |
| Ghir_A05G008920 | | Ghir_A09G009700 | 0.05 | | Ghir_A12G017120 | | Ghir_D05G008910 | | -0.18 |
| Ghir_A05G008930 | | Ghir_A09G009700 | -0.10 | | Ghir_A13G009000 | | Ghir_D05G008910 | | -0.07 |
| Ghir_A09G009680 | | Ghir_A09G009700 | 0.71 | | Ghir_A13G023660 | | Ghir_D05G008910 | | -0.22 |
| Ghir_A03G021820 | | Ghir_A12G004130 | 0.58 | | Ghir_A03G021820 | | Ghir_D07G011260 | | -0.26 |
| Ghir_A05G008920 | | Ghir_A12G004130 | -0.19 | | Ghir_A05G008920 | | Ghir_D07G011260 | | -0.09 |
| Ghir_A05G008930 | | Ghir_A12G004130 | -0.10 | | Ghir_A05G008930 | | Ghir_D07G011260 | | 0.14 |
| Ghir_A09G009680 | | Ghir_A12G004130 | -0.08 | | Ghir_A09G009680 | | Ghir_D07G011260 | | -0.07 |
| Ghir_A09G009700 | | Ghir_A12G004130 | 0.20 | | Ghir_A09G009700 | | Ghir_D07G011260 | | -0.20 |
| Ghir_A03G021820 | | Ghir_A12G008140 | 0.14 | | Ghir_A12G004130 | | Ghir_D07G011260 | | -0.04 |
| Ghir_A05G008920 | | Ghir_A12G008140 | 0.03 | | Ghir_A12G008140 | | Ghir_D07G011260 | | -0.28 |
| Ghir_A05G008930 | | Ghir_A12G008140 | -0.12 | | Ghir_A12G017120 | | Ghir_D07G011260 | | 0.03 |
| Ghir_A09G009680 | | Ghir_A12G008140 | -0.10 | | Ghir_A13G009000 | | Ghir_D07G011260 | | 0.16 |
| Ghir_A09G009700 | | Ghir_A12G008140 | -0.15 | | Ghir_A13G023660 | | Ghir_D07G011260 | | -0.19 |
| Ghir_A12G004130 | | Ghir_A12G008140 | -0.12 | | Ghir_D05G008910 | | Ghir_D07G011260 | | -0.09 |
| Ghir_A03G021820 | | Ghir_A12G017120 | 0.01 | | Ghir_A03G021820 | | Ghir_D09G009410 | | 0.11 |
| Ghir_A05G008920 | | Ghir_A12G017120 | -0.18 | | Ghir_A05G008920 | | Ghir_D09G009410 | | 0.00 |
| Ghir_A05G008930 | | Ghir_A12G017120 | 0.09 | | Ghir_A05G008930 | | Ghir_D09G009410 | | -0.13 |
| Ghir_A09G009680 | | Ghir_A12G017120 | -0.04 | | Ghir_A09G009680 | | Ghir_D09G009410 | | 0.73 |
| Ghir_A09G009700 | | Ghir_A12G017120 | -0.05 | | Ghir_A09G009700 | | Ghir_D09G009410 | | 0.92 |
| Ghir_A12G004130 | | Ghir_A12G017120 | 0.29 | | Ghir_A12G004130 | | Ghir_D09G009410 | | 0.09 |
| Ghir_A12G008140 | | Ghir_A12G017120 | -0.12 | | Ghir_A12G008140 | | Ghir_D09G009410 | | 0.01 |
| Ghir_A03G021820 | | Ghir_A13G009000 | 0.06 | | Ghir_A12G017120 | | Ghir_D09G009410 | | -0.14 |
| Ghir_A05G008920 | | Ghir_A13G009000 | -0.07 | | Ghir_A13G009000 | | Ghir_D09G009410 | | -0.49 |
| Ghir_A05G008930 | | Ghir_A13G009000 | -0.06 | | Ghir_A13G023660 | | Ghir_D09G009410 | | -0.23 |
| Ghir_A09G009680 | | Ghir_A13G009000 | 0.00 | | Ghir_D05G008910 | | Ghir_D09G009410 | | 0.00 |
| Ghir_A09G009700 | | Ghir_A13G009000 | -0.05 | | Ghir_D07G011260 | | Ghir_D09G009410 | | -0.08 |
| Ghir_A12G004130 | | Ghir_A13G009000 | 0.06 | | Ghir_A03G021820 | | Ghir_D12G003150 | | 1.00 |
| Ghir_A12G008140 | | Ghir_A13G009000 | 0.00 | | Ghir_A05G008920 | | Ghir_D12G003150 | | -0.03 |
| Ghir_A12G017120 | | Ghir_A13G009000 | 0.73 | | Ghir_A05G008930 | | Ghir_D12G003150 | | -0.14 |
| Ghir_A03G021820 | | Ghir_A13G023660 | 0.00 | | Ghir_A09G009680 | | Ghir_D12G003150 | | -0.11 |
| Ghir_A05G008920 | | Ghir_A13G023660 | -0.22 | | Ghir_A09G009700 | | Ghir_D12G003150 | | 0.07 |
| Ghir_A05G008930 | | Ghir_A13G023660 | -0.05 | | Ghir_A12G004130 | | Ghir_D12G003150 | | 0.58 |
| Ghir_A09G009680 | | Ghir_A13G023660 | 0.11 | | Ghir_A12G008140 | | Ghir_D12G003150 | | 0.14 |
| Ghir_A09G009700 | | Ghir_A13G023660 | -0.04 | | Ghir_A12G017120 | | Ghir_D12G003150 | | 0.01 |
| Ghir_A12G004130 | | Ghir_A13G023660 | -0.13 | | Ghir_A13G009000 | | Ghir_D12G003150 | | 0.06 |
| Ghir_A12G008140 | | Ghir_A13G023660 | 0.01 | | Ghir_A13G023660 | | Ghir_D12G003150 | | 0.00 |
| Ghir_A12G017120 | | Ghir_A13G023660 | 0.14 | | Ghir_D05G008910 | | Ghir_D12G003150 | | -0.03 |
| Ghir_A13G009000 | | Ghir_A13G023660 | 0.20 | | Ghir_D07G011260 | | Ghir_D12G003150 | | -0.26 |
| Ghir_D09G009410 | | Ghir_D12G003150 | 0.11 | | Ghir_A05G008920 | | Ghir_D13G024390 | | -0.09 |
| Ghir_A03G021820 | | Ghir_D12G007600 | 0.28 | | Ghir_A05G008930 | | Ghir_D13G024390 | | 0.05 |
| Ghir_A05G008920 | | Ghir_D12G007600 | -0.14 | | Ghir_A09G009680 | | Ghir_D13G024390 | | -0.06 |
| Ghir_A05G008930 | | Ghir_D12G007600 | -0.03 | | Ghir_A09G009700 | | Ghir_D13G024390 | | -0.03 |
| Ghir_A09G009680 | | Ghir_D12G007600 | -0.32 | | Ghir_A12G004130 | | Ghir_D13G024390 | | -0.06 |
| Ghir_A09G009700 | | Ghir_D12G007600 | -0.02 | | Ghir_A12G008140 | | Ghir_D13G024390 | | 0.20 |
| Ghir_A12G004130 | | Ghir_D12G007600 | 0.39 | | Ghir_A12G017120 | | Ghir_D13G024390 | | -0.01 |
| Ghir_A12G008140 | | Ghir_D12G007600 | 1.00 | | Ghir_A13G009000 | | Ghir_D13G024390 | | 0.09 |
| Ghir_A12G017120 | | Ghir_D12G007600 | 0.10 | | Ghir_A13G023660 | | Ghir_D13G024390 | | 1.00 |
| Ghir_A13G009000 | | Ghir_D12G007600 | 0.02 | | Ghir_D05G008910 | | Ghir_D13G024390 | | -0.09 |
| Ghir_A13G023660 | | Ghir_D12G007600 | -0.04 | | Ghir_D07G011260 | | Ghir_D13G024390 | | 0.01 |
| Ghir_D05G008910 | | Ghir_D12G007600 | -0.14 | | Ghir_D09G009410 | | Ghir_D13G024390 | | -0.28 |
| Ghir_D07G011260 | | Ghir_D12G007600 | 0.01 | | Ghir_D12G003150 | | Ghir_D13G024390 | | -0.20 |
| Ghir_D09G009410 | | Ghir_D12G007600 | -0.02 | | Ghir_D12G007600 | | Ghir_D13G024390 | | 0.09 |
| Ghir_D12G003150 | | Ghir_D12G007600 | 0.28 | | Ghir_D12G017370 | | Ghir_D13G024390 | | -0.01 |
| Ghir_A03G021820 | | Ghir_D12G017370 | 0.01 | | Ghir_D13G010010 | | Ghir_D13G024390 | | -0.02 |
| Ghir_A05G008920 | | Ghir_D12G017370 | -0.18 | | Ghir_A03G021820 | | Ghir_A07G025290 | | -0.12 |
| Ghir_A05G008930 | | Ghir_D12G017370 | 0.09 | | Ghir_A05G008920 | | Ghir_A07G025290 | | -0.10 |
| Ghir_A09G009680 | | Ghir_D12G017370 | -0.04 | | Ghir_A05G008930 | | Ghir_A07G025290 | | -0.22 |
| Ghir_A09G009700 | | Ghir_D12G017370 | -0.05 | | Ghir_A09G009680 | | Ghir_A07G025290 | | 0.35 |
| Ghir_A12G004130 | | Ghir_D12G017370 | 0.29 | | Ghir_A09G009700 | | Ghir_A07G025290 | | 0.35 |
| Ghir_A12G008140 | | Ghir_D12G017370 | -0.12 | | Ghir_A12G004130 | | Ghir_A07G025290 | | 0.12 |
| Ghir_A12G017120 | | Ghir_D12G017370 | 1.00 | | Ghir_A12G008140 | | Ghir_A07G025290 | | -0.20 |
| Ghir_A13G009000 | | Ghir_D12G017370 | 0.73 | | Ghir_A12G017120 | | Ghir_A07G025290 | | -0.08 |
| Ghir_A13G023660 | | Ghir_D12G017370 | 0.14 | | Ghir_A13G009000 | | Ghir_A07G025290 | | 0.03 |
| Ghir_D05G008910 | | Ghir_D12G017370 | -0.18 | | Ghir_A13G023660 | | Ghir_A07G025290 | | -0.11 |
| Ghir_D07G011260 | | Ghir_D12G017370 | 0.03 | | Ghir_D05G008910 | | Ghir_A07G025290 | | -0.10 |
| Ghir_D09G009410 | | Ghir_D12G017370 | -0.14 | | Ghir_D07G011260 | | Ghir_A07G025290 | | 0.74 |
| Ghir_D12G003150 | | Ghir_D12G017370 | 0.01 | | Ghir_D09G009410 | | Ghir_A07G025290 | | 0.49 |
| Ghir_D12G007600 | | Ghir_D12G017370 | 0.10 | | Ghir_D12G003150 | | Ghir_A07G025290 | | -0.12 |
| Ghir_A03G021820 | | Ghir_D13G010010 | 0.05 | | Ghir_D12G007600 | | Ghir_A07G025290 | | 0.06 |
| Ghir_A05G008920 | | Ghir_D13G010010 | 0.12 | | Ghir_D12G017370 | | Ghir_A07G025290 | | -0.08 |
| Ghir_A05G008930 | | Ghir_D13G010010 | 0.45 | | Ghir_D13G010010 | | Ghir_A07G025290 | | -0.32 |
| Ghir_A09G009680 | | Ghir_D13G010010 | -0.06 | | Ghir_D13G024390 | | Ghir_A07G025290 | | -0.07 |
| Ghir_A09G009700 | | Ghir_D13G010010 | -0.20 | | Ghir_A03G021820 | | Ghir_D09G009430 | | -0.12 |
| Ghir_A12G004130 | | Ghir_D13G010010 | -0.15 | | Ghir_A05G008920 | | Ghir_D09G009430 | | 0.12 |
| Ghir_A12G008140 | | Ghir_D13G010010 | -0.03 | | Ghir_A05G008930 | | Ghir_D09G009430 | | 0.49 |
| Ghir_A12G017120 | | Ghir_D13G010010 | 0.08 | | Ghir_A09G009680 | | Ghir_D09G009430 | | 0.61 |
| Ghir_A13G009000 | | Ghir_D13G010010 | 0.49 | | Ghir_A09G009700 | | Ghir_D09G009430 | | 0.43 |
| Ghir_A13G023660 | | Ghir_D13G010010 | -0.11 | | Ghir_A12G004130 | | Ghir_D09G009430 | | 0.00 |
| Ghir_D05G008910 | | Ghir_D13G010010 | 0.12 | | Ghir_A12G008140 | | Ghir_D09G009430 | | -0.15 |
| Ghir_D07G011260 | | Ghir_D13G010010 | 0.23 | | Ghir_A12G017120 | | Ghir_D09G009430 | | 0.00 |
| Ghir_D09G009410 | | Ghir_D13G010010 | -0.05 | | Ghir_A13G009000 | | Ghir_D09G009430 | | -0.19 |
| Ghir_D12G003150 | | Ghir_D13G010010 | 0.05 | | Ghir_A13G023660 | | Ghir_D09G009430 | | -0.20 |
| Ghir_D12G007600 | | Ghir_D13G010010 | -0.17 | | Ghir_D05G008910 | | Ghir_D09G009430 | | 0.12 |
| Ghir_D12G017370 | | Ghir_D13G010010 | 0.08 | | Ghir_D07G011260 | | Ghir_D09G009430 | | 0.20 |
| Ghir_A03G021820 | | Ghir_D13G024390 | -0.20 | | Ghir_D09G009410 | | Ghir_D09G009430 | | 0.17 |
| Ghir_D12G017370 | | Ghir_D09G009430 | 0.00 | | Ghir_D12G003150 | | Ghir_D09G009430 | | -0.12 |
| Ghir_D13G010010 | | Ghir_D09G009430 | 0.08 | | Ghir_D12G007600 | | Ghir_D09G009430 | | -0.33 |
| Ghir_D13G024390 | | Ghir_D09G009430 | -0.05 | | Ghir_A07G025290 | | Ghir_D09G009430 | | 0.07 |
| **Reproductive organs** | | |  | |  | |  | |  |
| **Source gene** | **Target gene** | | **Pcor.** | | **Source gene** | | **Target gene** | | **Pcor.** |
| Ghir_A03G021820 | Ghir_A05G008920 | | 0.01 | | Ghir_A03G021820 | | Ghir_D05G008910 | | 0.00 |
| Ghir_A03G021820 | Ghir_A05G008930 | | -0.08 | | Ghir_A05G008920 | | Ghir_D05G008910 | | 1.00 |
| Ghir_A05G008920 | Ghir_A05G008930 | | 0.03 | | Ghir_A05G008930 | | Ghir_D05G008910 | | 0.03 |
| Ghir_A03G021820 | Ghir_A09G009680 | | 0.03 | | Ghir_A09G009680 | | Ghir_D05G008910 | | -0.10 |
| Ghir_A05G008920 | Ghir_A09G009680 | | -0.10 | | Ghir_A09G009700 | | Ghir_D05G008910 | | 0.00 |
| Ghir_A05G008930 | Ghir_A09G009680 | | -0.09 | | Ghir_A12G004130 | | Ghir_D05G008910 | | -0.03 |
| Ghir_A03G021820 | Ghir_A09G009700 | | -0.06 | | Ghir_A12G008140 | | Ghir_D05G008910 | | 0.08 |
| Ghir_A05G008920 | Ghir_A09G009700 | | 0.00 | | Ghir_A12G017120 | | Ghir_D05G008910 | | 0.17 |
| Ghir_A05G008930 | Ghir_A09G009700 | | 0.01 | | Ghir_A13G009000 | | Ghir_D05G008910 | | -0.06 |
| Ghir_A09G009680 | Ghir_A09G009700 | | 1.00 | | Ghir_A13G023660 | | Ghir_D05G008910 | | -0.11 |
| Ghir_A03G021820 | Ghir_A12G004130 | | 0.08 | | Ghir_A03G021820 | | Ghir_D07G011260 | | -0.08 |
| Ghir_A05G008920 | Ghir_A12G004130 | | -0.03 | | Ghir_A05G008920 | | Ghir_D07G011260 | | -0.11 |
| Ghir_A05G008930 | Ghir_A12G004130 | | -0.17 | | Ghir_A05G008930 | | Ghir_D07G011260 | | -0.11 |
| Ghir_A09G009680 | Ghir_A12G004130 | | 0.08 | | Ghir_A09G009680 | | Ghir_D07G011260 | | 0.11 |
| Ghir_A09G009700 | Ghir_A12G004130 | | 0.65 | | Ghir_A09G009700 | | Ghir_D07G011260 | | 0.10 |
| Ghir_A03G021820 | Ghir_A12G008140 | | -0.10 | | Ghir_A12G004130 | | Ghir_D07G011260 | | -0.03 |
| Ghir_A05G008920 | Ghir_A12G008140 | | 0.08 | | Ghir_A12G008140 | | Ghir_D07G011260 | | 0.20 |
| Ghir_A05G008930 | Ghir_A12G008140 | | -0.03 | | Ghir_A12G017120 | | Ghir_D07G011260 | | 0.01 |
| Ghir_A09G009680 | Ghir_A12G008140 | | -0.11 | | Ghir_A13G009000 | | Ghir_D07G011260 | | 0.00 |
| Ghir_A09G009700 | Ghir_A12G008140 | | -0.03 | | Ghir_A13G023660 | | Ghir_D07G011260 | | 0.18 |
| Ghir_A12G004130 | Ghir_A12G008140 | | -0.36 | | Ghir_D05G008910 | | Ghir_D07G011260 | | -0.11 |
| Ghir_A03G021820 | Ghir_A12G017120 | | -0.02 | | Ghir_A03G021820 | | Ghir_D09G009410 | | 0.01 |
| Ghir_A05G008920 | Ghir_A12G017120 | | 0.17 | | Ghir_A05G008920 | | Ghir_D09G009410 | | 0.02 |
| Ghir_A05G008930 | Ghir_A12G017120 | | -0.26 | | Ghir_A05G008930 | | Ghir_D09G009410 | | -0.13 |
| Ghir_A09G009680 | Ghir_A12G017120 | | -0.05 | | Ghir_A09G009680 | | Ghir_D09G009410 | | 0.19 |
| Ghir_A09G009700 | Ghir_A12G017120 | | -0.02 | | Ghir_A09G009700 | | Ghir_D09G009410 | | 0.05 |
| Ghir_A12G004130 | Ghir_A12G017120 | | -0.22 | | Ghir_A12G004130 | | Ghir_D09G009410 | | -0.31 |
| Ghir_A12G008140 | Ghir_A12G017120 | | 0.28 | | Ghir_A12G008140 | | Ghir_D09G009410 | | 0.05 |
| Ghir_A03G021820 | Ghir_A13G009000 | | 0.98 | | Ghir_A12G017120 | | Ghir_D09G009410 | | 0.17 |
| Ghir_A05G008920 | Ghir_A13G009000 | | -0.06 | | Ghir_A13G009000 | | Ghir_D09G009410 | | 0.04 |
| Ghir_A05G008930 | Ghir_A13G009000 | | 0.04 | | Ghir_A13G023660 | | Ghir_D09G009410 | | -0.13 |
| Ghir_A09G009680 | Ghir_A13G009000 | | -0.13 | | Ghir_D05G008910 | | Ghir_D09G009410 | | 0.02 |
| Ghir_A09G009700 | Ghir_A13G009000 | | -0.01 | | Ghir_D07G011260 | | Ghir_D09G009410 | | 1.00 |
| Ghir_A12G004130 | Ghir_A13G009000 | | -0.07 | | Ghir_A03G021820 | | Ghir_D12G003150 | | 1.00 |
| Ghir_A12G008140 | Ghir_A13G009000 | | -0.03 | | Ghir_A05G008920 | | Ghir_D12G003150 | | 0.00 |
| Ghir_A12G017120 | Ghir_A13G009000 | | -0.02 | | Ghir_A05G008930 | | Ghir_D12G003150 | | -0.08 |
| Ghir_A03G021820 | Ghir_A13G023660 | | -0.06 | | Ghir_A09G009680 | | Ghir_D12G003150 | | 0.03 |
| Ghir_A05G008920 | Ghir_A13G023660 | | -0.11 | | Ghir_A09G009700 | | Ghir_D12G003150 | | -0.06 |
| Ghir_A05G008930 | Ghir_A13G023660 | | -0.23 | | Ghir_A12G004130 | | Ghir_D12G003150 | | 0.08 |
| Ghir_A09G009680 | Ghir_A13G023660 | | -0.03 | | Ghir_A12G008140 | | Ghir_D12G003150 | | -0.10 |
| Ghir_A09G009700 | Ghir_A13G023660 | | 0.19 | | Ghir_A12G017120 | | Ghir_D12G003150 | | -0.02 |
| Ghir_A12G004130 | Ghir_A13G023660 | | 0.06 | | Ghir_A13G009000 | | Ghir_D12G003150 | | 0.98 |
| Ghir_A12G008140 | Ghir_A13G023660 | | -0.04 | | Ghir_A13G023660 | | Ghir_D12G003150 | | -0.06 |
| Ghir_A12G017120 | Ghir_A13G023660 | | 0.02 | | Ghir_D05G008910 | | Ghir_D12G003150 | | 0.00 |
| Ghir_A13G009000 | Ghir_A13G023660 | | -0.09 | | Ghir_D07G011260 | | Ghir_D12G003150 | | -0.08 |
| Ghir_A05G008920 | Ghir_D13G024390 | | -0.10 | | Ghir_D09G009410 | | Ghir_D12G003150 | | 0.01 |
| Ghir_A05G008930 | Ghir_D13G024390 | | -0.19 | | Ghir_A03G021820 | | Ghir_D12G007600 | | -0.14 |
| Ghir_A09G009680 | Ghir_D13G024390 | | 0.32 | | Ghir_A05G008920 | | Ghir_D12G007600 | | -0.10 |
| Ghir_A09G009700 | Ghir_D13G024390 | | -0.06 | | Ghir_A05G008930 | | Ghir_D12G007600 | | 0.20 |
| Ghir_A12G004130 | Ghir_D13G024390 | | -0.20 | | Ghir_A09G009680 | | Ghir_D12G007600 | | -0.02 |
| Ghir_A12G008140 | Ghir_D13G024390 | | -0.22 | | Ghir_A09G009700 | | Ghir_D12G007600 | | 0.02 |
| Ghir_A12G017120 | Ghir_D13G024390 | | 0.11 | | Ghir_A12G004130 | | Ghir_D12G007600 | | -0.23 |
| Ghir_A13G009000 | Ghir_D13G024390 | | -0.21 | | Ghir_A12G008140 | | Ghir_D12G007600 | | 1.00 |
| Ghir_A13G023660 | Ghir_D13G024390 | | 0.49 | | Ghir_A12G017120 | | Ghir_D12G007600 | | 0.05 |
| Ghir_D05G008910 | Ghir_D13G024390 | | -0.10 | | Ghir_A13G009000 | | Ghir_D12G007600 | | 0.04 |
| Ghir_D07G011260 | Ghir_D13G024390 | | -0.15 | | Ghir_A13G023660 | | Ghir_D12G007600 | | -0.09 |
| Ghir_D09G009410 | Ghir_D13G024390 | | 0.10 | | Ghir_D05G008910 | | Ghir_D12G007600 | | -0.10 |
| Ghir_D12G003150 | Ghir_D13G024390 | | 0.05 | | Ghir_D07G011260 | | Ghir_D12G007600 | | 0.24 |
| Ghir_D12G007600 | Ghir_D13G024390 | | -0.18 | | Ghir_D09G009410 | | Ghir_D12G007600 | | 0.16 |
| Ghir_D12G017370 | Ghir_D13G024390 | | 0.11 | | Ghir_D12G003150 | | Ghir_D12G007600 | | -0.14 |
| Ghir_D13G010010 | Ghir_D13G024390 | | -0.14 | | Ghir_A03G021820 | | Ghir_D12G017370 | | -0.02 |
| Ghir_A03G021820 | Ghir_A07G025290 | | -0.01 | | Ghir_A05G008920 | | Ghir_D12G017370 | | 0.17 |
| Ghir_A05G008920 | Ghir_A07G025290 | | -0.09 | | Ghir_A05G008930 | | Ghir_D12G017370 | | -0.26 |
| Ghir_A05G008930 | Ghir_A07G025290 | | -0.13 | | Ghir_A09G009680 | | Ghir_D12G017370 | | -0.05 |
| Ghir_A09G009680 | Ghir_A07G025290 | | 0.56 | | Ghir_A09G009700 | | Ghir_D12G017370 | | -0.02 |
| Ghir_A09G009700 | Ghir_A07G025290 | | 0.38 | | Ghir_A12G004130 | | Ghir_D12G017370 | | -0.22 |
| Ghir_A12G004130 | Ghir_A07G025290 | | 0.37 | | Ghir_A12G008140 | | Ghir_D12G017370 | | 0.28 |
| Ghir_A12G008140 | Ghir_A07G025290 | | 0.11 | | Ghir_A12G017120 | | Ghir_D12G017370 | | 1.00 |
| Ghir_A12G017120 | Ghir_A07G025290 | | -0.08 | | Ghir_A13G009000 | | Ghir_D12G017370 | | -0.02 |
| Ghir_A13G009000 | Ghir_A07G025290 | | -0.10 | | Ghir_A13G023660 | | Ghir_D12G017370 | | 0.02 |
| Ghir_A13G023660 | Ghir_A07G025290 | | 0.22 | | Ghir_D05G008910 | | Ghir_D12G017370 | | 0.17 |
| Ghir_D05G008910 | Ghir_A07G025290 | | -0.09 | | Ghir_D07G011260 | | Ghir_D12G017370 | | 0.01 |
| Ghir_D07G011260 | Ghir_A07G025290 | | 0.23 | | Ghir_D09G009410 | | Ghir_D12G017370 | | 0.17 |
| Ghir_D09G009410 | Ghir_A07G025290 | | -0.15 | | Ghir_D12G003150 | | Ghir_D12G017370 | | -0.02 |
| Ghir_D12G003150 | Ghir_A07G025290 | | -0.01 | | Ghir_D12G007600 | | Ghir_D12G017370 | | 0.05 |
| Ghir_D12G007600 | Ghir_A07G025290 | | 0.04 | | Ghir_A03G021820 | | Ghir_D13G010010 | | 1.00 |
| Ghir_D12G017370 | Ghir_A07G025290 | | -0.08 | | Ghir_A05G008920 | | Ghir_D13G010010 | | 0.11 |
| Ghir_D13G010010 | Ghir_A07G025290 | | -0.07 | | Ghir_A05G008930 | | Ghir_D13G010010 | | -0.08 |
| Ghir_D13G024390 | Ghir_A07G025290 | | -0.11 | | Ghir_A09G009680 | | Ghir_D13G010010 | | -0.15 |
| Ghir_A03G021820 | Ghir_D09G009430 | | 0.04 | | Ghir_A09G009700 | | Ghir_D13G010010 | | -0.02 |
| Ghir_A05G008920 | Ghir_D09G009430 | | 0.28 | | Ghir_A12G004130 | | Ghir_D13G010010 | | 0.03 |
| Ghir_A05G008930 | Ghir_D09G009430 | | -0.20 | | Ghir_A12G008140 | | Ghir_D13G010010 | | 0.00 |
| Ghir_A09G009680 | Ghir_D09G009430 | | 0.93 | | Ghir_A12G017120 | | Ghir_D13G010010 | | 0.10 |
| Ghir_A09G009700 | Ghir_D09G009430 | | 1.00 | | Ghir_A13G009000 | | Ghir_D13G010010 | | 1.00 |
| Ghir_A12G004130 | Ghir_D09G009430 | | 0.05 | | Ghir_A13G023660 | | Ghir_D13G010010 | | -0.04 |
| Ghir_A12G008140 | Ghir_D09G009430 | | 0.08 | | Ghir_D05G008910 | | Ghir_D13G010010 | | 0.11 |
| Ghir_A12G017120 | Ghir_D09G009430 | | 0.17 | | Ghir_D07G011260 | | Ghir_D13G010010 | | -0.07 |
| Ghir_A13G009000 | Ghir_D09G009430 | | -0.07 | | Ghir_D09G009410 | | Ghir_D13G010010 | | -0.04 |
| Ghir_A13G023660 | Ghir_D09G009430 | | 0.10 | | Ghir_D12G003150 | | Ghir_D13G010010 | | 1.00 |
| Ghir_D05G008910 | Ghir_D09G009430 | | 0.28 | | Ghir_D12G007600 | | Ghir_D13G010010 | | -0.20 |
| Ghir_D07G011260 | Ghir_D09G009430 | | 0.11 | | Ghir_D12G017370 | | Ghir_D13G010010 | | 0.10 |
| Ghir_D09G009410 | Ghir_D09G009430 | | 0.33 | | Ghir_A03G021820 | | Ghir_D13G024390 | | 0.05 |
| Ghir_D12G003150 | Ghir_D09G009430 | | 0.04 | | Ghir_D13G010010 | | Ghir_D09G009430 | | 0.08 |
| Ghir_D12G007600 | Ghir_D09G009430 | | -0.09 | | Ghir_D13G024390 | | Ghir_D09G009430 | | 0.02 |
| Ghir_D12G017370 | Ghir_D09G009430 | | 0.17 | | Ghir_A07G025290 | | Ghir_D09G009430 | | -0.01 |
| **Fiber Development** | | |  |  | |  | |  | |
| **Source gene** | **Target gene** | | **Pcor.** | **Source gene** | | **Target gene** | | **Pcor.** | |
| Ghir_A03G021820 | Ghir_A05G008920 | | 0.50 | Ghir_A05G008920 | | Ghir_D05G008910 | | 0.59 | |
| Ghir_A03G021820 | Ghir_A05G008930 | | 0.44 | Ghir_A05G008930 | | Ghir_D05G008910 | | 0.23 | |
| Ghir_A05G008920 | Ghir_A05G008930 | | 0.23 | Ghir_A09G009680 | | Ghir_D05G008910 | | -0.49 | |
| Ghir_A03G021820 | Ghir_A09G009680 | | -0.36 | Ghir_A09G009700 | | Ghir_D05G008910 | | 0.04 | |
| Ghir_A05G008920 | Ghir_A09G009680 | | -0.49 | Ghir_A12G004130 | | Ghir_D05G008910 | | 0.36 | |
| Ghir_A05G008930 | Ghir_A09G009680 | | 0.07 | Ghir_A12G008140 | | Ghir_D05G008910 | | 0.31 | |
| Ghir_A03G021820 | Ghir_A09G009700 | | 0.06 | Ghir_A12G017120 | | Ghir_D05G008910 | | 0.17 | |
| Ghir_A05G008920 | Ghir_A09G009700 | | 0.04 | Ghir_A13G009000 | | Ghir_D05G008910 | | 0.40 | |
| Ghir_A05G008930 | Ghir_A09G009700 | | 0.04 | Ghir_A13G023660 | | Ghir_D05G008910 | | -0.29 | |
| Ghir_A09G009680 | Ghir_A09G009700 | | 0.38 | Ghir_A03G021820 | | Ghir_D07G011260 | | -0.70 | |
| Ghir_A03G021820 | Ghir_A12G004130 | | 0.31 | Ghir_A05G008920 | | Ghir_D07G011260 | | -0.43 | |
| Ghir_A05G008920 | Ghir_A12G004130 | | 0.36 | Ghir_A05G008930 | | Ghir_D07G011260 | | -0.68 | |
| Ghir_A05G008930 | Ghir_A12G004130 | | 0.04 | Ghir_A09G009680 | | Ghir_D07G011260 | | 0.42 | |
| Ghir_A09G009680 | Ghir_A12G004130 | | -0.26 | Ghir_A09G009700 | | Ghir_D07G011260 | | 0.04 | |
| Ghir_A09G009700 | Ghir_A12G004130 | | 0.58 | Ghir_A12G004130 | | Ghir_D07G011260 | | -0.25 | |
| Ghir_A03G021820 | Ghir_A12G008140 | | 0.33 | Ghir_A12G008140 | | Ghir_D07G011260 | | -0.50 | |
| Ghir_A05G008920 | Ghir_A12G008140 | | 0.31 | Ghir_A12G017120 | | Ghir_D07G011260 | | 0.18 | |
| Ghir_A05G008930 | Ghir_A12G008140 | | 0.37 | Ghir_A13G009000 | | Ghir_D07G011260 | | -0.58 | |
| Ghir_A09G009680 | Ghir_A12G008140 | | -0.62 | Ghir_A13G023660 | | Ghir_D07G011260 | | 0.28 | |
| Ghir_A09G009700 | Ghir_A12G008140 | | -0.56 | Ghir_D05G008910 | | Ghir_D07G011260 | | -0.43 | |
| Ghir_A12G004130 | Ghir_A12G008140 | | -0.14 | Ghir_A03G021820 | | Ghir_D09G009410 | | -0.32 | |
| Ghir_A03G021820 | Ghir_A12G017120 | | 0.00 | Ghir_A05G008920 | | Ghir_D09G009410 | | -0.25 | |
| Ghir_A05G008920 | Ghir_A12G017120 | | 0.17 | Ghir_A05G008930 | | Ghir_D09G009410 | | -0.46 | |
| Ghir_A05G008930 | Ghir_A12G017120 | | -0.57 | Ghir_A09G009680 | | Ghir_D09G009410 | | -0.16 | |
| Ghir_A09G009680 | Ghir_A12G017120 | | -0.32 | Ghir_A09G009700 | | Ghir_D09G009410 | | -0.68 | |
| Ghir_A09G009700 | Ghir_A12G017120 | | 0.38 | Ghir_A12G004130 | | Ghir_D09G009410 | | -0.60 | |
| Ghir_A12G004130 | Ghir_A12G017120 | | 0.55 | Ghir_A12G008140 | | Ghir_D09G009410 | | 0.21 | |
| Ghir_A12G008140 | Ghir_A12G017120 | | -0.27 | Ghir_A12G017120 | | Ghir_D09G009410 | | -0.16 | |
| Ghir_A03G021820 | Ghir_A13G009000 | | 0.41 | Ghir_A13G009000 | | Ghir_D09G009410 | | 0.11 | |
| Ghir_A05G008920 | Ghir_A13G009000 | | 0.40 | Ghir_A13G023660 | | Ghir_D09G009410 | | 0.78 | |
| Ghir_A05G008930 | Ghir_A13G009000 | | 0.39 | Ghir_D05G008910 | | Ghir_D09G009410 | | -0.25 | |
| Ghir_A09G009680 | Ghir_A13G009000 | | -0.71 | Ghir_D07G011260 | | Ghir_D09G009410 | | 0.35 | |
| Ghir_A09G009700 | Ghir_A13G009000 | | -0.48 | Ghir_A03G021820 | | Ghir_D12G003150 | | 0.62 | |
| Ghir_A12G004130 | Ghir_A13G009000 | | 0.00 | Ghir_A05G008920 | | Ghir_D12G003150 | | 0.50 | |
| Ghir_A12G008140 | Ghir_A13G009000 | | 0.98 | Ghir_A05G008930 | | Ghir_D12G003150 | | 0.44 | |
| Ghir_A12G017120 | Ghir_A13G009000 | | -0.18 | Ghir_A09G009680 | | Ghir_D12G003150 | | -0.36 | |
| Ghir_A03G021820 | Ghir_A13G023660 | | -0.29 | Ghir_A09G009700 | | Ghir_D12G003150 | | 0.06 | |
| Ghir_A05G008920 | Ghir_A13G023660 | | -0.29 | Ghir_A12G004130 | | Ghir_D12G003150 | | 0.31 | |
| Ghir_A05G008930 | Ghir_A13G023660 | | -0.21 | Ghir_A12G008140 | | Ghir_D12G003150 | | 0.33 | |
| Ghir_A09G009680 | Ghir_A13G023660 | | 0.08 | Ghir_A12G017120 | | Ghir_D12G003150 | | 0.00 | |
| Ghir_A09G009700 | Ghir_A13G023660 | | -0.58 | Ghir_A13G009000 | | Ghir_D12G003150 | | 0.41 | |
| Ghir_A12G004130 | Ghir_A13G023660 | | -0.82 | Ghir_A13G023660 | | Ghir_D12G003150 | | -0.29 | |
| Ghir_A12G008140 | Ghir_A13G023660 | | 0.15 | Ghir_D05G008910 | | Ghir_D12G003150 | | 0.50 | |
| Ghir_A12G017120 | Ghir_A13G023660 | | -0.35 | Ghir_D07G011260 | | Ghir_D12G003150 | | -0.70 | |
| Ghir_A13G009000 | Ghir_A13G023660 | | 0.04 | Ghir_D09G009410 | | Ghir_D12G003150 | | -0.32 | |
| Ghir_A03G021820 | Ghir_D05G008910 | | 0.50 | Ghir_A03G021820 | | Ghir_D12G007600 | | 0.36 | |
| Ghir_A05G008930 | Ghir_D13G024390 | | -0.17 | Ghir_D12G003150 | | Ghir_D13G024390 | | -0.44 | |
| Ghir_A09G009680 | Ghir_D13G024390 | | 0.53 | Ghir_D12G007600 | | Ghir_D13G024390 | | -0.15 | |
| Ghir_A09G009700 | Ghir_D13G024390 | | -0.13 | Ghir_D12G017370 | | Ghir_D13G024390 | | -0.30 | |
| Ghir_A12G004130 | Ghir_D13G024390 | | -0.47 | Ghir_D13G010010 | | Ghir_D13G024390 | | -0.15 | |
| Ghir_A12G008140 | Ghir_D13G024390 | | -0.26 | Ghir_A03G021820 | | Ghir_A07G025290 | | 0.17 | |
| Ghir_A12G017120 | Ghir_D13G024390 | | -0.30 | Ghir_A05G008920 | | Ghir_A07G025290 | | 0.18 | |
| Ghir_A13G009000 | Ghir_D13G024390 | | -0.37 | Ghir_A05G008930 | | Ghir_A07G025290 | | 0.07 | |
| Ghir_A13G023660 | Ghir_D13G024390 | | 0.38 | Ghir_A09G009680 | | Ghir_A07G025290 | | 0.07 | |
| Ghir_D05G008910 | Ghir_D13G024390 | | -0.62 | Ghir_A09G009700 | | Ghir_A07G025290 | | 0.79 | |
| Ghir_D07G011260 | Ghir_D13G024390 | | 0.42 | Ghir_A12G004130 | | Ghir_A07G025290 | | 0.72 | |
| Ghir_D09G009410 | Ghir_D13G024390 | | 0.31 | Ghir_A12G008140 | | Ghir_A07G025290 | | -0.31 | |
| Ghir_A03G021820 | Ghir_D09G009430 | | -0.48 | Ghir_A12G004130 | | Ghir_D09G009430 | | -0.34 | |
| Ghir_A05G008920 | Ghir_D09G009430 | | -0.60 | Ghir_A12G008140 | | Ghir_D09G009430 | | -0.36 | |
| Ghir_A05G008930 | Ghir_D09G009430 | | -0.21 | Ghir_A12G017120 | | Ghir_D09G009430 | | -0.18 | |
| Ghir_A09G009680 | Ghir_D09G009430 | | 0.55 | Ghir_A13G009000 | | Ghir_D09G009430 | | -0.45 | |
| Ghir_A09G009700 | Ghir_D09G009430 | | 0.02 | Ghir_A13G023660 | | Ghir_D09G009430 | | 0.26 | |
| Ghir_A05G008920 | Ghir_D12G007600 | | 0.24 | Ghir_A09G009680 | | Ghir_D13G010010 | | -0.76 | |
| Ghir_A05G008930 | Ghir_D12G007600 | | 0.68 | Ghir_A09G009700 | | Ghir_D13G010010 | | -0.85 | |
| Ghir_A09G009680 | Ghir_D12G007600 | | -0.38 | Ghir_A12G004130 | | Ghir_D13G010010 | | -0.31 | |
| Ghir_A09G009700 | Ghir_D12G007600 | | -0.54 | Ghir_A12G008140 | | Ghir_D13G010010 | | 0.69 | |
| Ghir_A12G004130 | Ghir_D12G007600 | | -0.26 | Ghir_A12G017120 | | Ghir_D13G010010 | | -0.11 | |
| Ghir_A12G008140 | Ghir_D12G007600 | | 0.83 | Ghir_A13G009000 | | Ghir_D13G010010 | | 0.68 | |
| Ghir_A12G017120 | Ghir_D12G007600 | | -0.57 | Ghir_A13G023660 | | Ghir_D13G010010 | | 0.40 | |
| Ghir_A13G009000 | Ghir_D12G007600 | | 0.74 | Ghir_D05G008910 | | Ghir_D13G010010 | | 0.19 | |
| Ghir_A13G023660 | Ghir_D12G007600 | | 0.17 | Ghir_D07G011260 | | Ghir_D13G010010 | | -0.20 | |
| Ghir_D05G008910 | Ghir_D12G007600 | | 0.24 | Ghir_D09G009410 | | Ghir_D13G010010 | | 0.60 | |
| Ghir_D07G011260 | Ghir_D12G007600 | | -0.60 | Ghir_D12G003150 | | Ghir_D13G010010 | | 0.11 | |
| Ghir_D09G009410 | Ghir_D12G007600 | | 0.11 | Ghir_D12G007600 | | Ghir_D13G010010 | | 0.55 | |
| Ghir_D12G003150 | Ghir_D12G007600 | | 0.36 | Ghir_D12G017370 | | Ghir_D13G010010 | | -0.11 | |
| Ghir_A03G021820 | Ghir_D12G017370 | | 0.00 | Ghir_A03G021820 | | Ghir_D13G024390 | | -0.44 | |
| Ghir_A05G008920 | Ghir_D12G017370 | | 0.17 | Ghir_A05G008920 | | Ghir_D13G024390 | | -0.62 | |
| Ghir_A05G008930 | Ghir_D12G017370 | | -0.57 | Ghir_A12G017120 | | Ghir_A07G025290 | | 0.40 | |
| Ghir_A09G009680 | Ghir_D12G017370 | | -0.32 | Ghir_A13G009000 | | Ghir_A07G025290 | | -0.22 | |
| Ghir_A09G009700 | Ghir_D12G017370 | | 0.38 | Ghir_A13G023660 | | Ghir_A07G025290 | | -0.75 | |
| Ghir_A12G004130 | Ghir_D12G017370 | | 0.55 | Ghir_D05G008910 | | Ghir_A07G025290 | | 0.18 | |
| Ghir_A12G008140 | Ghir_D12G017370 | | -0.27 | Ghir_D07G011260 | | Ghir_A07G025290 | | -0.11 | |
| Ghir_A12G017120 | Ghir_D12G017370 | | 1.00 | Ghir_D09G009410 | | Ghir_A07G025290 | | -0.70 | |
| Ghir_A13G009000 | Ghir_D12G017370 | | -0.18 | Ghir_D12G003150 | | Ghir_A07G025290 | | 0.17 | |
| Ghir_A13G023660 | Ghir_D12G017370 | | -0.35 | Ghir_D12G007600 | | Ghir_A07G025290 | | -0.34 | |
| Ghir_D05G008910 | Ghir_D12G017370 | | 0.17 | Ghir_D12G017370 | | Ghir_A07G025290 | | 0.40 | |
| Ghir_D07G011260 | Ghir_D12G017370 | | 0.18 | Ghir_D13G010010 | | Ghir_A07G025290 | | -0.51 | |
| Ghir_D09G009410 | Ghir_D12G017370 | | -0.16 | Ghir_D13G024390 | | Ghir_A07G025290 | | -0.27 | |
| Ghir_D12G003150 | Ghir_D12G017370 | | 0.00 | Ghir_D05G008910 | | Ghir_D09G009430 | | -0.60 | |
| Ghir_D12G007600 | Ghir_D12G017370 | | -0.57 | Ghir_D07G011260 | | Ghir_D09G009430 | | 0.44 | |
| Ghir_A03G021820 | Ghir_D13G010010 | | 0.11 | Ghir_D09G009410 | | Ghir_D09G009430 | | 0.19 | |
| Ghir_A05G008920 | Ghir_D13G010010 | | 0.19 | Ghir_D12G003150 | | Ghir_D09G009430 | | -0.48 | |
| Ghir_A05G008930 | Ghir_D13G010010 | | -0.10 | Ghir_D12G007600 | | Ghir_D09G009430 | | -0.28 | |
| **Nitrogen metabolism** | | |  |  | |  | |  | |
| **Source gene** | **Target gene** | | **Pcor.** | **Source gene** | | **Target gene** | | **Pcor.** | |
| Ghir_A03G021820 | Ghir_A05G008920 | | 0.65 | Ghir_A03G021820 | | Ghir_D05G008910 | | 0.39 | |
| Ghir_A03G021820 | Ghir_A05G008930 | | 0.31 | Ghir_A05G008920 | | Ghir_D05G008910 | | 0.41 | |
| Ghir_A05G008920 | Ghir_A05G008930 | | 0.00 | Ghir_A05G008930 | | Ghir_D05G008910 | | -0.32 | |
| Ghir_A03G021820 | Ghir_A09G009680 | | 0.30 | Ghir_A09G009680 | | Ghir_D05G008910 | | 0.46 | |
| Ghir_A05G008920 | Ghir_A09G009680 | | 0.30 | Ghir_A09G009700 | | Ghir_D05G008910 | | 0.70 | |
| Ghir_A05G008930 | Ghir_A09G009680 | | -0.31 | Ghir_A12G004130 | | Ghir_D05G008910 | | 0.28 | |
| Ghir_A03G021820 | Ghir_A09G009700 | | 0.39 | Ghir_A12G008140 | | Ghir_D05G008910 | | 0.50 | |
| Ghir_A05G008920 | Ghir_A09G009700 | | 0.41 | Ghir_A12G017120 | | Ghir_D05G008910 | | 0.34 | |
| Ghir_A05G008930 | Ghir_A09G009700 | | -0.33 | Ghir_A13G009000 | | Ghir_D05G008910 | | 0.01 | |
| Ghir_A09G009680 | Ghir_A09G009700 | | 0.46 | Ghir_A13G023660 | | Ghir_D05G008910 | | 0.61 | |
| Ghir_A03G021820 | Ghir_A12G004130 | | 0.34 | Ghir_A03G021820 | | Ghir_D07G011260 | | 0.33 | |
| Ghir_A05G008920 | Ghir_A12G004130 | | 0.29 | Ghir_A05G008920 | | Ghir_D07G011260 | | 0.30 | |
| Ghir_A05G008930 | Ghir_A12G004130 | | -0.16 | Ghir_A05G008930 | | Ghir_D07G011260 | | -0.23 | |
| Ghir_A09G009680 | Ghir_A12G004130 | | 0.41 | Ghir_A09G009680 | | Ghir_D07G011260 | | 0.54 | |
| Ghir_A09G009700 | Ghir_A12G004130 | | 0.28 | Ghir_A09G009700 | | Ghir_D07G011260 | | 0.34 | |
| Ghir_A03G021820 | Ghir_A12G008140 | | 0.42 | Ghir_A12G004130 | | Ghir_D07G011260 | | 0.50 | |
| Ghir_A05G008920 | Ghir_A12G008140 | | 0.53 | Ghir_A12G008140 | | Ghir_D07G011260 | | 0.31 | |
| Ghir_A05G008930 | Ghir_A12G008140 | | -0.08 | Ghir_A12G017120 | | Ghir_D07G011260 | | 0.36 | |
| Ghir_A09G009680 | Ghir_A12G008140 | | 0.36 | Ghir_A13G009000 | | Ghir_D07G011260 | | 0.11 | |
| Ghir_A09G009700 | Ghir_A12G008140 | | 0.50 | Ghir_A13G023660 | | Ghir_D07G011260 | | 0.40 | |
| Ghir_A12G004130 | Ghir_A12G008140 | | 0.25 | Ghir_D05G008910 | | Ghir_D07G011260 | | 0.33 | |
| Ghir_A03G021820 | Ghir_A12G017120 | | 0.67 | Ghir_A03G021820 | | Ghir_D09G009410 | | -0.40 | |
| Ghir_A05G008920 | Ghir_A12G017120 | | 0.60 | Ghir_A05G008920 | | Ghir_D09G009410 | | -0.35 | |
| Ghir_A05G008930 | Ghir_A12G017120 | | 0.04 | Ghir_A05G008930 | | Ghir_D09G009410 | | 0.07 | |
| Ghir_A09G009680 | Ghir_A12G017120 | | 0.31 | Ghir_A09G009680 | | Ghir_D09G009410 | | -0.42 | |
| Ghir_A09G009700 | Ghir_A12G017120 | | 0.35 | Ghir_A09G009700 | | Ghir_D09G009410 | | -0.29 | |
| Ghir_A12G004130 | Ghir_A12G017120 | | 0.33 | Ghir_A12G004130 | | Ghir_D09G009410 | | -0.53 | |
| Ghir_A12G008140 | Ghir_A12G017120 | | 0.47 | Ghir_A12G008140 | | Ghir_D09G009410 | | -0.26 | |
| Ghir_A03G021820 | Ghir_A13G009000 | | 0.66 | Ghir_A12G017120 | | Ghir_D09G009410 | | -0.46 | |
| Ghir_A05G008920 | Ghir_A13G009000 | | 0.32 | Ghir_A13G009000 | | Ghir_D09G009410 | | -0.26 | |
| Ghir_A05G008930 | Ghir_A13G009000 | | 0.80 | Ghir_A13G023660 | | Ghir_D09G009410 | | -0.28 | |
| Ghir_A09G009680 | Ghir_A13G009000 | | 0.03 | Ghir_D05G008910 | | Ghir_D09G009410 | | -0.29 | |
| Ghir_A09G009700 | Ghir_A13G009000 | | 0.01 | Ghir_D07G011260 | | Ghir_D09G009410 | | -0.50 | |
| Ghir_A12G004130 | Ghir_A13G009000 | | 0.20 | Ghir_A03G021820 | | Ghir_D12G003150 | | -0.71 | |
| Ghir_A12G008140 | Ghir_A13G009000 | | 0.19 | Ghir_A05G008920 | | Ghir_D12G003150 | | -0.43 | |
| Ghir_A12G017120 | Ghir_A13G009000 | | 0.37 | Ghir_A05G008930 | | Ghir_D12G003150 | | -0.47 | |
| Ghir_A03G021820 | Ghir_A13G023660 | | 0.33 | Ghir_A09G009680 | | Ghir_D12G003150 | | -0.26 | |
| Ghir_A05G008920 | Ghir_A13G023660 | | 0.37 | Ghir_A09G009700 | | Ghir_D12G003150 | | -0.19 | |
| Ghir_A05G008930 | Ghir_A13G023660 | | -0.31 | Ghir_A12G004130 | | Ghir_D12G003150 | | -0.44 | |
| Ghir_A09G009680 | Ghir_A13G023660 | | 0.50 | Ghir_A12G008140 | | Ghir_D12G003150 | | -0.30 | |
| Ghir_A09G009700 | Ghir_A13G023660 | | 0.62 | Ghir_A12G017120 | | Ghir_D12G003150 | | -0.50 | |
| Ghir_A12G004130 | Ghir_A13G023660 | | 0.28 | Ghir_A13G009000 | | Ghir_D12G003150 | | -0.90 | |
| Ghir_A12G008140 | Ghir_A13G023660 | | 0.45 | Ghir_A13G023660 | | Ghir_D12G003150 | | -0.20 | |
| Ghir_A12G017120 | Ghir_A13G023660 | | 0.31 | Ghir_D05G008910 | | Ghir_D12G003150 | | -0.19 | |
| Ghir_A13G009000 | Ghir_A13G023660 | | 0.01 | Ghir_D07G011260 | | Ghir_D12G003150 | | -0.33 | |
| Ghir_A05G008920 | Ghir_D13G024390 | | -0.49 | Ghir_D09G009410 | | Ghir_D12G003150 | | 0.45 | |
| Ghir_A05G008930 | Ghir_D13G024390 | | 0.04 | Ghir_A03G021820 | | Ghir_D12G007600 | | -0.15 | |
| Ghir_A09G009680 | Ghir_D13G024390 | | -0.40 | Ghir_A05G008920 | | Ghir_D12G007600 | | -0.27 | |
| Ghir_A09G009700 | Ghir_D13G024390 | | -0.31 | Ghir_A05G008930 | | Ghir_D12G007600 | | 0.67 | |
| Ghir_A12G004130 | Ghir_D13G024390 | | -0.35 | Ghir_A09G009680 | | Ghir_D12G007600 | | -0.32 | |
| Ghir_A12G008140 | Ghir_D13G024390 | | -0.41 | Ghir_A09G009700 | | Ghir_D12G007600 | | -0.55 | |
| Ghir_A12G017120 | Ghir_D13G024390 | | -0.52 | Ghir_A12G004130 | | Ghir_D12G007600 | | -0.08 | |
| Ghir_A13G009000 | Ghir_D13G024390 | | -0.25 | Ghir_A12G008140 | | Ghir_D12G007600 | | -0.32 | |
| Ghir_A13G023660 | Ghir_D13G024390 | | -0.34 | Ghir_A12G017120 | | Ghir_D12G007600 | | -0.18 | |
| Ghir_D05G008910 | Ghir_D13G024390 | | -0.31 | Ghir_A13G009000 | | Ghir_D12G007600 | | 0.45 | |
| Ghir_D07G011260 | Ghir_D13G024390 | | -0.43 | Ghir_A13G023660 | | Ghir_D12G007600 | | -0.44 | |
| Ghir_D09G009410 | Ghir_D13G024390 | | 0.47 | Ghir_D05G008910 | | Ghir_D12G007600 | | -0.54 | |
| Ghir_D12G003150 | Ghir_D13G024390 | | 0.39 | Ghir_D07G011260 | | Ghir_D12G007600 | | -0.24 | |
| Ghir_D12G007600 | Ghir_D13G024390 | | 0.17 | Ghir_D09G009410 | | Ghir_D12G007600 | | 0.11 | |
| Ghir_D12G017370 | Ghir_D13G024390 | | 0.31 | Ghir_D12G003150 | | Ghir_D12G007600 | | -0.33 | |
| Ghir_D13G010010 | Ghir_D13G024390 | | -0.31 | Ghir_A03G021820 | | Ghir_D12G017370 | | -0.31 | |
| Ghir_A03G021820 | Ghir_A07G025290 | | 0.41 | Ghir_A05G008920 | | Ghir_D12G017370 | | -0.28 | |
| Ghir_A05G008920 | Ghir_A07G025290 | | 0.15 | Ghir_A05G008930 | | Ghir_D12G017370 | | 0.22 | |
| Ghir_A05G008930 | Ghir_A07G025290 | | 0.74 | Ghir_A09G009680 | | Ghir_D12G017370 | | -0.37 | |
| Ghir_A09G009680 | Ghir_A07G025290 | | -0.23 | Ghir_A09G009700 | | Ghir_D12G017370 | | -0.27 | |
| Ghir_A09G009700 | Ghir_A07G025290 | | -0.03 | Ghir_A12G004130 | | Ghir_D12G017370 | | -0.66 | |
| Ghir_A12G004130 | Ghir_A07G025290 | | -0.25 | Ghir_A12G008140 | | Ghir_D12G017370 | | -0.23 | |
| Ghir_A12G008140 | Ghir_A07G025290 | | 0.09 | Ghir_A12G017120 | | Ghir_D12G017370 | | -0.33 | |
| Ghir_A12G017120 | Ghir_A07G025290 | | 0.10 | Ghir_A13G009000 | | Ghir_D12G017370 | | -0.18 | |
| Ghir_A13G009000 | Ghir_A07G025290 | | 0.56 | Ghir_A13G023660 | | Ghir_D12G017370 | | -0.28 | |
| Ghir_A13G023660 | Ghir_A07G025290 | | -0.10 | Ghir_D05G008910 | | Ghir_D12G017370 | | -0.27 | |
| Ghir_D05G008910 | Ghir_A07G025290 | | -0.03 | Ghir_D07G011260 | | Ghir_D12G017370 | | -0.44 | |
| Ghir_D07G011260 | Ghir_A07G025290 | | -0.20 | Ghir_D09G009410 | | Ghir_D12G017370 | | 0.48 | |
| Ghir_D09G009410 | Ghir_A07G025290 | | 0.10 | Ghir_D12G003150 | | Ghir_D12G017370 | | 0.49 | |
| Ghir_D12G003150 | Ghir_A07G025290 | | -0.18 | Ghir_D12G007600 | | Ghir_D12G017370 | | 0.00 | |
| Ghir_D12G007600 | Ghir_A07G025290 | | 0.01 | Ghir_A03G021820 | | Ghir_D13G010010 | | 0.33 | |
| Ghir_D12G017370 | Ghir_A07G025290 | | 0.41 | Ghir_A05G008920 | | Ghir_D13G010010 | | 0.29 | |
| Ghir_D13G010010 | Ghir_A07G025290 | | -0.38 | Ghir_A05G008930 | | Ghir_D13G010010 | | -0.17 | |
| Ghir_D13G024390 | Ghir_A07G025290 | | 0.00 | Ghir_A09G009680 | | Ghir_D13G010010 | | 0.36 | |
| Ghir_A03G021820 | Ghir_D09G009430 | | 0.73 | Ghir_A09G009700 | | Ghir_D13G010010 | | 0.26 | |
| Ghir_A05G008920 | Ghir_D09G009430 | | 0.47 | Ghir_A12G004130 | | Ghir_D13G010010 | | 0.66 | |
| Ghir_A05G008930 | Ghir_D09G009430 | | 0.41 | Ghir_A12G008140 | | Ghir_D13G010010 | | 0.23 | |
| Ghir_A09G009680 | Ghir_D09G009430 | | 0.14 | Ghir_A12G017120 | | Ghir_D13G010010 | | 0.34 | |
| Ghir_A09G009700 | Ghir_D09G009430 | | 0.37 | Ghir_A13G009000 | | Ghir_D13G010010 | | 0.23 | |
| Ghir_A12G004130 | Ghir_D09G009430 | | 0.08 | Ghir_A13G023660 | | Ghir_D13G010010 | | 0.28 | |
| Ghir_A12G008140 | Ghir_D09G009430 | | 0.39 | Ghir_D05G008910 | | Ghir_D13G010010 | | 0.26 | |
| Ghir_A12G017120 | Ghir_D09G009430 | | 0.42 | Ghir_D07G011260 | | Ghir_D13G010010 | | 0.43 | |
| Ghir_A13G009000 | Ghir_D09G009430 | | 0.58 | Ghir_D09G009410 | | Ghir_D13G010010 | | -0.49 | |
| Ghir_A13G023660 | Ghir_D09G009430 | | 0.27 | Ghir_D12G003150 | | Ghir_D13G010010 | | -0.52 | |
| Ghir_D05G008910 | Ghir_D09G009430 | | 0.37 | Ghir_D12G007600 | | Ghir_D13G010010 | | 0.03 | |
| Ghir_D07G011260 | Ghir_D09G009430 | | 0.15 | Ghir_D12G017370 | | Ghir_D13G010010 | | -0.79 | |
| Ghir_D09G009410 | Ghir_D09G009430 | | -0.21 | Ghir_A03G021820 | | Ghir_D13G024390 | | -0.40 | |
| Ghir_D12G003150 | Ghir_D09G009430 | | -0.42 | Ghir_D13G010010 | | Ghir_D09G009430 | | -0.03 | |
| Ghir_D12G007600 | Ghir_D09G009430 | | -0.37 | Ghir_D13G024390 | | Ghir_D09G009430 | | -0.29 | |
| Ghir_D12G017370 | Ghir_D09G009430 | | 0.05 | Ghir_A07G025290 | | Ghir_D09G009430 | | 0.81 | |
